# FGIN-1-27 Inhibits Melanogenesis by Regulating Protein Kinase A/cAMP-Responsive Element-Binding, Protein Kinase C-β, and Mitogen-Activated Protein Kinase Pathways

**DOI:** 10.3389/fphar.2020.602889

**Published:** 2020-12-03

**Authors:** Jinpeng Lv, Songzhou Jiang, Ying Yang, Ximei Zhang, Rongyin Gao, Yan Cao, Guoqiang Song

**Affiliations:** ^1^ College of Pharmaceutical Engineering and Life Sciences, Changzhou University, Changzhou, China; ^2^ Department of Pharmacy, The First People’s Hospital of Changzhou, The third Affiliated Hospital of Soochow University, Changzhou, China; ^3^ Department of Dermatology, The Affiliated Changzhou No. 2 People’s Hospital of Nanjing Medical University, Changzhou, China

**Keywords:** FGIN-1-27, human epidermis melanocytes, SK-MEL-2 cells, melanogenesis, protein kinase A/cAMP-responsive element-binding, protein kinase C-β, mitogen-activated protein kinase

## Abstract

FGIN-1-27 is a synthetic mitochondrial diazepam binding inhibitor receptor (MDR) agonist that has demonstrated pro-apoptotic, anti-anxiety, and steroidogenic activity in various studies. Here we report, for the first time, the anti-melanogenic efficacy of FGIN-1-27 *in vitro* and *in vivo*. FGIN-1-27 significantly inhibited basal and α-melanocyte-stimulating hormone (α-MSH)-, 1-Oleoyl-2-acetyl-sn-glycerol (OAG)- and Endothelin-1 (ET-1)-induced melanogenesis without cellular toxicity. Mushroom tyrosinase activity assay showed that FGIN-1-27 did not directly inhibit tyrosinase activity, which suggested that FGIN-1-27 was not a direct inhibitor of tyrosinase. Although it was not capable of modulating the catalytic activity of mushroom tyrosinase *in vitro*, FGIN-1-27 downregulated the expression levels of key proteins that function in melanogenesis. FGIN-1-27 played these functions mainly by suppressing the PKA/CREB, PKC-β, and MAPK pathways. Once inactivated, it decreased the expression of MITF, tyrosinase, TRP-1, TRP-2, and inhibited the tyrosinase activity, finally inhibiting melanogenesis. During *in vivo* experiments, FGIN-1-27 inhibited the body pigmentation of zebrafish and reduced UVB-induced hyperpigmentation in guinea pig skin, but not a reduction of numbers of melanocytes. Our findings indicated that FGIN-1-27 exhibited no cytotoxicity and inhibited melanogenesis in both *in vitro* and *in vivo* models. It may prove quite useful as a safer skin-whitening agent.

## Introduction

Melanogenesis is the most important function of melanocytes in the skin. Several critical factors involving in melanogenesis have been clarified (Raposo and Marks, 2007; Noguchi et al., 2014). The key enzyme that regulates melanogenesis is tyrosinase and it acts with tyrosinase-related protein 1 (TRP1) and tyrosinase-related protein 2 (TRP2) to promote melanin synthesis (Zhu et al., 2015). Another important factor involved in pigmentation is microphthalmia-associated transcription factor (MITF), which not only regulates the proliferation and survival of melanocytes but also regulates tyrosinase, TRP1 and TRP2 expression (Kawasaki et al., 2008; Levy and Fisher, 2011). Environmental stimuli, such as ultraviolet (UV) irradiation, play a crucial role in melanogenesis (Abdel-Naser et al., 2003). On exposure to UV radiation, activated keratinocytes and melanocytes produce α-melanocyte-stimulating hormone (α-MSH), diacylglycerol (DAG), and endothelin-1 (ET-1) (D'Mello et al., 2016; Bae-Harboe and Park, 2012). When α-melanocyte-stimulating hormone (α-MSH) binds to melanocortin-1 receptor (MC1R) in melanocytes, it could increase the intracellular levels of cyclic adenosine monophosphate (cAMP) and activate the protein kinase A (PKA)/ (CREB) pathway, finally promoting melanogenesis (Corre et al., 2004; Rzepka et al., 2016). Diacylglycerol (DAG) is essential for the activation of protein kinase C-β (PKC-β) in melanocytes, which increases the activity of tyrosinase and induces pigmentation (Kim et al., 2006; Kawaguchi et al., 2012; Yuan and Jin, 2018). 1-Oleoyl-2-acetyl-sn-glycerol (OAG) is a synthetic, membrane-permeable DAG analog have been used extensively as pharmacological activators of PKC-β (Rainbow et al., 2011). Endothelin-1 (ET-1) induces melanogenesis in melanocytes via the activation of mitogen-activated protein kinase (MAPK) pathway (Park et al., 2015; Regazzetti et al., 2015).

Melanin plays an important role in the protection of skin from the harmful effects of ultraviolet radiation, such as skin cancer and aging (Slominski et al., 2004). Nevertheless, excessive production and abnormal accumulation of melanin lead to skin esthetic troubles, such as melasma, freckles, and dark spots (Jadotte and Schwartz, 2010). Several of the active whitening compounds, including kojic acid and arbutin, have already been approved as cosmetic additives (Kim et al., 2008). But it has been reported that arbutin has cytotoxic effects and kojic acid can induce cancer (Singh et al., 2016). Thus, the discovery of more efficient and safer skin-whitening agents is paramount.

Mitochondrial diazepam binding inhibitor receptor (MDR) plays a central role in the regulation of lipid and steroid synthesis and is widely distributed in peripheral tissues including skin (Alho et al., 1993). In our previous studies, diazepam, one MDR agonist, regulated pigmentation via the PKA/CREB pathway (Lv et al., 2019). Furthermore, recent findings suggested that MDR activation slightly increased the number of new melanocytes in zebrafish (Allen et al., 2020). In comparison with diazepam, FGIN-1-27 (N, N-Dihexyl-2-(4-fluorophenyl)indole-3-acetamide) exhibits a relatively higher affinity and specificity for the MDR (Freeman and Young, 2000). FGIN-1–27 has demonstrated pro-apoptotic and steroidogenic activity (Lacapère and Papadopoulos, 2003). Recently, it has been reported that FGIN-1-27 produced anti-anxiety and anti-panic effects *in vivo* (Lima-Maximino et al., 2018). However, the effect of FGIN-1-27 on pigmentation *in vitro* and *in vivo* has not been reported.

Here we examined whether FGIN-1-27 would affect melanogenesis. The melanogenesis-related parameters, such as the expression of MITF, tyrosinase, TRP-1, and TRP-2, the cellular tyrosinase activity, and the PKA/CREB, PKC-β and MAPK pathway were also investigated to elucidate how FGIN-1-27 regulate melanogenesis. Finally, the anti-melanogenic effects of FGIN-1-27 were confirmed *in vivo* in zebrafish and in UV-induced hyperpigmentation in brown guinea pigs.

## Materials and Methods

### Materials

FGIN-1-27(N,N-Dihexyl-2-(4-fluorophenyl)indole-3-acetamide), OAG (1-Oleoyl-2-acetyl-sn-glycerol) and the antibodies against PKC-β (1:200), p-PKA cat (phospho T197) (1:200), PKA cat (1:500), p-CREB (1:200), CREB(1:200), p-ERK (1:200), ERK (1:200), p-p38 (1:200), p38 (1:200), p-JNK (1:200), and JNK (1:200) were obtained from Santa Cruz Biotechnology (CA, USA). Tyrosinase from mushroom, α-MSH, ET-1(Endothelin-1), and PTU(N-Phenylthiourea) were obtained from Aladdin (Shanghai, China). The antibodies against tyrosinase (1:1,000), TRP-1(1:1,000), TRP-2 (1:1,000), MITF (1:2000), and β-actin (1:3,000) were obtained from Abcam (Cambridge, UK). Masson-Fontana melanin staining solution was purchased from SenBeiJia Biological Technology (Nanjing, China). RT-qPCR kits were purchased from Takara Biomedical Technology (Beijing, China). Cell lysis buffer and BCA protein assay kit were obtained from Beyotime Biotechnology (Shanghai, China).

### Cells Culture

The human melanotic melanoma cell line, SK-MEL-2, was provided by the Chinese Academy of Sciences (Shanghai, China). SK-MEL-2 was maintained in the DMEM (GIBCO, USA) supplemented with 10% FBS (HyClone, USA), 100 U/ml penicillin and 100 μg/ml streptomycin (GIBCO, USA). Human *epidermis* melanocytes (HEM) from ScienCell Research Laboratories (CA, USA) is isolated from neonatal human skin. HEM was cultured in the Medium 254 (GIBCO, USA) added with Human Melanocyte Growth Supplement (GIBCO, USA), 100 U/ml penicillin and 100 μg/ml streptomycin (GIBCO, USA). All of these cells were maintained in a humidified atmosphere containing 5% CO_2_ at 37°C.

### Cell Viability Assay

The viability of SK-MEL-2 cells and HEM were measured through the MTT assay (Zhou et al., 2014). SK-MEL-2 cells and HEM were respectively plated in 96-well plates at a density of 2×10^4^ cells per well. FGIN-1-27 (0, 1, 2, 4, 8, 16 μM) was applied to SK-MEL-2 cells and HEM for 48 h. MTT solution (20 μL) was added to each well of a 96-well plate for another 4 h. After removing the solution, DMSO (200 μL) was added to each well and the absorbance was examined at 570 nm using a microplate spectrophotometer (BioTek Instruments).

### Melanin Assay

SK-MEL-2 cells and HEM were respectively plated in 6-well plates at a concentration of 5×10^5^ cells per well. After 24 h incubation at 37°C, FGIN-1-27 (0, 1, 2, 4 μM) was applied to SK-MEL-2 cells and HEM. The cells were incubated for another 48 h, washed with PBS, followed by lysis with cell lysis buffer for 20 min at 4°C, and the lysates were centrifuged at 13,000 rpm for 10 min at 4°C. The total melanin in the cell pellet was dissolved in 100 μL NaOH solution (1 mol/L, containing 10% DMSO) at 80°C for 1 h (Lv et al., 2015). Optical absorbance was measured at 405 nm using a microplate spectrophotometer (BioTek Instruments).

### Tyrosinase Activity Assay

The cellular tyrosinase activity was conducted as previously described (Lv et al., 2020). SK-MEL-2 cells were incubated with different concentrations of FGIN-1-27 (0, 1, 2, 4 μM) or OAG (200 μM) for 12 h or 48 h and then lysed with cell lysis buffer. Cell lysates were centrifuged at 13,000 rpm for 10 min at 4°C to obtain the supernatant for tyrosinase activity assay. Protein concentrations were determined by BCA kit with bovine serum albumin (BSA) as a standard.100 μL PBS (0.1 M, pH 6.5) containing 30 μg proteins mixed with 100 μL L-DOPA (0.1%) and then incubated at 37°C for 1 h. Optical absorbance was measured at 475 nm using a microplate spectrophotometer (BioTek Instruments).

The direct effect of FGIN-1-27 on tyrosinase activity was conducted in accordance with a previous method (Tomita et al., 2010; Lv et al., 2020). 100 μL PBS (0.1 M, pH 6.5) containing different concentrations of FGIN-1-27 mixed with mushroom tyrosinase (10 unit) and 50 μL L-tyrosine (0.05%) and then incubated at 37°C for 10 min. Optical absorbance was measured at 475 nm using a microplate spectrophotometer (BioTek Instruments).

### Masson–Fontana Ammoniacal Silver Staining

For the detection of melanin pigment, SK-MEL-2 cells were plated on a 6-well plate containing 13-mm glass coverslips in 2.0 ml of culture medium (10% fetal bovine serum) in each well. At 48 h after the administration, cells were fixed with Formalin (10% neutral buffered) for 20 min.

Cells and skin slides were stained according to the Masson–Fontana ammoniacal silver staining method (Lee et al., 2013). Cells and skin slides were incubated with Masson-Fontana melanin staining solution for 16 h at room temperature. Then cells and skin slides were rinsed in distilled water 3 times and were incubated with hypo solution for 7 min. Following thorough washing in distilled water, cells and skin slides were counterstained with neutral red stain for 7 min. Cells and skin slides were observed using a Nikon Ti2-U microscope.

### Immunohistochemistry for S-100

S-100 immunohistochemistry was conducted as previously described (Lee et al., 2013). Slides were blocked with 5% BSA for 1 h at room temperature and then incubated in anti-S-100 primary antibody (Dako, Carpentaria, CA) diluted in blocking solution overnight at 4°C. Next day, Slides were washed with TBST 4 times, incubated with secondary antibody (Dako, Carpentaria, CA). Then, slides were treated with aminoethylcarbazole to develop the sections. Skin slides were observed using a Nikon Ti2-U microscope.

### Reverse Transcription-Quantitative PCR (RT-qPCR)

RT-qPCR was conducted as previously described (Lv et al., 2020). Briefly, SK-MEL-2 cell total RNA was extracted using TRIzol reagent and quantified spectrophotometrically. Single-stranded cDNA was synthesized using SuperScript II Reverse transcriptase according to the manufacturer’s instructions. SYBR-Green quantitative PCR analysis was performed with an ABI PRISM Sequence Detection System (Applied Biosystems) and pre-validated primer sets. All samples were run in triplicate. Threshold cycles were placed in the logarithmic portion of the amplification curve, and the results were normalized to GAPDH. The fold difference between two samples was determined by the delta-delta Cq method. The primers sequences of Mitf gene are 5′-AGA​GCA​GGG​CAG​AGA​GTG​AGT​G-3′, 5′-AAC​TTG​ATT​CCA​GGC​TGA​TGA​TGT​C-3′.

### Western Blot Analysis

The protein suspension was obtained as the method mentioned above. The extracted intracellular proteins (30 μg) were separated by SDS-PAGE (10%) and then transferred to a polyvinylidene fluoride (PVDF) membrane, following a wet transfer standard protocol. The membrane was blocked using 2.5% BSA at room temperature for 1 h, exposed to appropriate primary antibodies at 4°C overnight and washed with TBST 4 times. The membranes were exposed to HRP-labeled Goat Anti-Rabbit IgG (1:1,000) or Goat Anti-Mouse IgG (1:1,000) at room temperature for 1 h and washed with TBST 4 times (Liao et al., 2017). The membranes were then visualized using a multifunctional chemiluminescence imaging system (Tanon 4600). Internal controls from the same blot were probed with anti-β actin.

### Phenotype-Based Evaluation and Determination of Melanin Content in Zebrafish

Adult zebrafishes were kept in a tank with the following conditions: 28.5°C, with a 14/10 h light/dark cycle. Embryos were obtained from natural spawning that was induced in the morning by turning on the light. The collection of embryos was completed within 30 min. Phenotype-based evaluation was conducted as described previously (Choi et al., 2007). Briefly, synchronized embryos were collected and arrayed by pipette (3-4 embryos per well in 96-well plates containing 200 μl of embryo medium). FGIN-1-27 and PTU were dissolved in 0.1% DMSO, then added to the embryo medium from 35 to 60 h (25 h exposure). The effects on the melanogenesis of zebrafish were observed under the stereomicroscope.

### UVB-Induced Hyperpigmentation Guinea Pig Model

Eight brown guinea pigs (6 weeks, approximately 300 g) were obtained from the Institute of Laboratory Animal Science (Beijing, China). The guinea pigs were housed individually in a temperature-controlled room under standard laboratory conditions, with a 12 h light/12 h dark cycle (lights on 9:00 AM to 9:00 PM) with constant temperature (23 ± 3°C) and humidity (50 ± 10%). The separate areas (1 cm diametrical circle) of the back of each animal were exposed to 500 mJ/cm^2^ UVB (Sigma SH-4, Shanghai, China) once a day for 1 week (Fujimoto et al., 1988; Lee et al., 2013). Then the vehicle (PEG400/EtOH = 7:3) and FGIN-1-27 (1%) were given to the hyperpigmented areas (20 μl solution per circle) twice a day for 3 weeks. The degree of pigmentation was measured by the Spectrophotometer (YS3010, 3nh, Shenzhen, China). The Δ*L*-value was determined according to the L-value measured by the spectrophotometer, as follows: Δ*L* = *L* (at each day measured)-*L* (at day 0). All animal procedures for this study were conducted according to the “NIH Guide for the Care and Use of Laboratory Animals” and approved by the animal care and use committee of Changzhou University.

### Statistical Analysis

Data are presented as mean ± SEM. The statistical analysis was performed using one-way ANOVA, followed by Turkey’s post hoc test for multiple comparison tests. All statistical calculations were performed using GraphPad Prism 7.0. A *p* value less than 0.05 is considered significant.

## Results

### FGIN-1-27 Inhibited α-MSH, OAG or ET-1 -Induced Melanin Synthesis in SK-MEL-2 Cells

**FIGURE 1:**
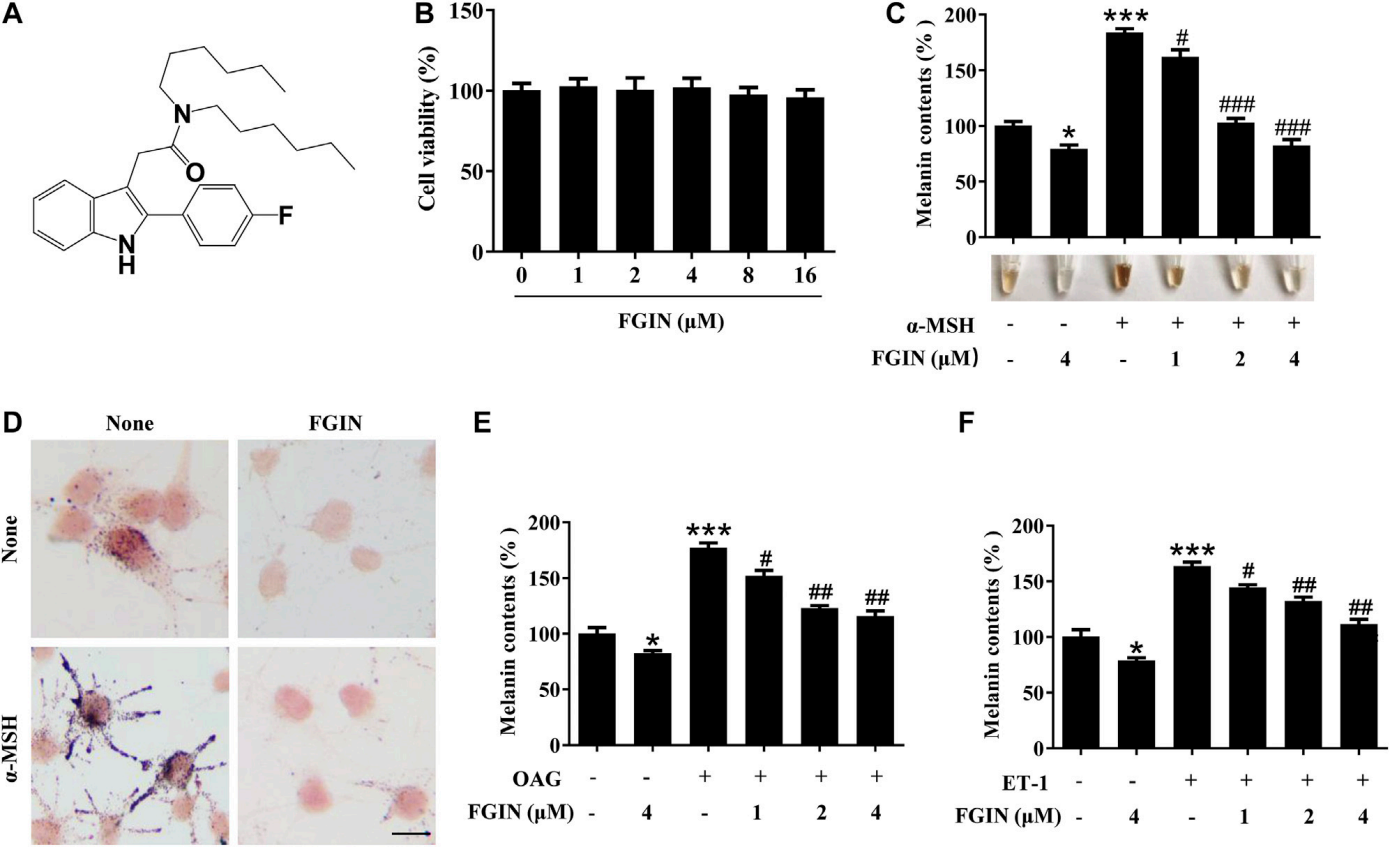
Effect of FGIN-1-27 (FGIN) on melanogenesis in SK-MEL-2 cells. **(A)** The chemical structure of FGIN-1-27, **(B)** after incubation of with various concentrations (1–16 μM) of FGIN-1-27 for 48 h, cell viability was determined using MTT assay, **(C)** SK-MEL-2 cells were treated with FGIN-1-27 in the presence or absence of α-MSH (50 nM) for 48 h. Melanin contents were measured as described in methods. **(D)** SK-MEL-2 cells were treated with FGIN-1-27 (4 μM) for 48 h and were stained with Masson–Fontana ammoniacal silver stain. Bar = 20 μm **(E,F)** SK-MEL-2 cells were treated with 200 μM OAG or 10 nM ET-1 in the presence or absence of FGIN-1-27. Melanin contents were measured as described in methods. Data are expressed as the mean ± SD (*n* = 3). *p < 0.05, ***p < 0.001 vs. non-treated cells. ^#^ *p* < 0.05, ^##^ *p* < 0.01, ^###^ *p* < 0.001 vs. α-MSH-, OAG-, or ET-1-treated cells.

Before investigating the effects of FGIN-1-27 (Figure 1A) on pigmentation, cell viability assay was performed to examine the effects of FGIN-1-27 on SK-MEL-2 cell growth. As shown in Figure 1B, FGIN-1-27 had no effects on the growth of SK-MEL-2 cells at a dosing range of 1–16 μM after 48 h. In melanin content assay, FGIN-1-27 inhibited the basal melanogenesis (Figure 1C). α-MSH is an elevator of intracellular cAMP, which significantly induces melanogenesis (Rzepka et al., 2016; Corre et al., 2004; Lee et al., 2013). FGIN-1-27 also inhibited α-MSH-induced melanin synthesis (Figure 1C). Consistently, Masson–Fontana ammoniacal silver staining indicated that FGIN-1-27 significantly decreased the melanin concentration in SK-MEL-2 cells with or without α-MSH (Figure 1D). Furthermore, OAG is an elevator of PKC and ET-1 is an activator of the MAPK pathway, both of which can promote melanin synthesis (Kim et al., 2006; Park et al., 2015; Regazzetti et al., 2015). As shown in Figure 1E and 1F, FGIN-1-27 also markedly inhibited OAG or ET-1-induced melanogenesis.

### FGIN-1-27 Suppressed Tyrosinase, TRP-1, TRP-2 Expression and Decreased the Cellular Tyrosinase Activity

**FIGURE 2:**
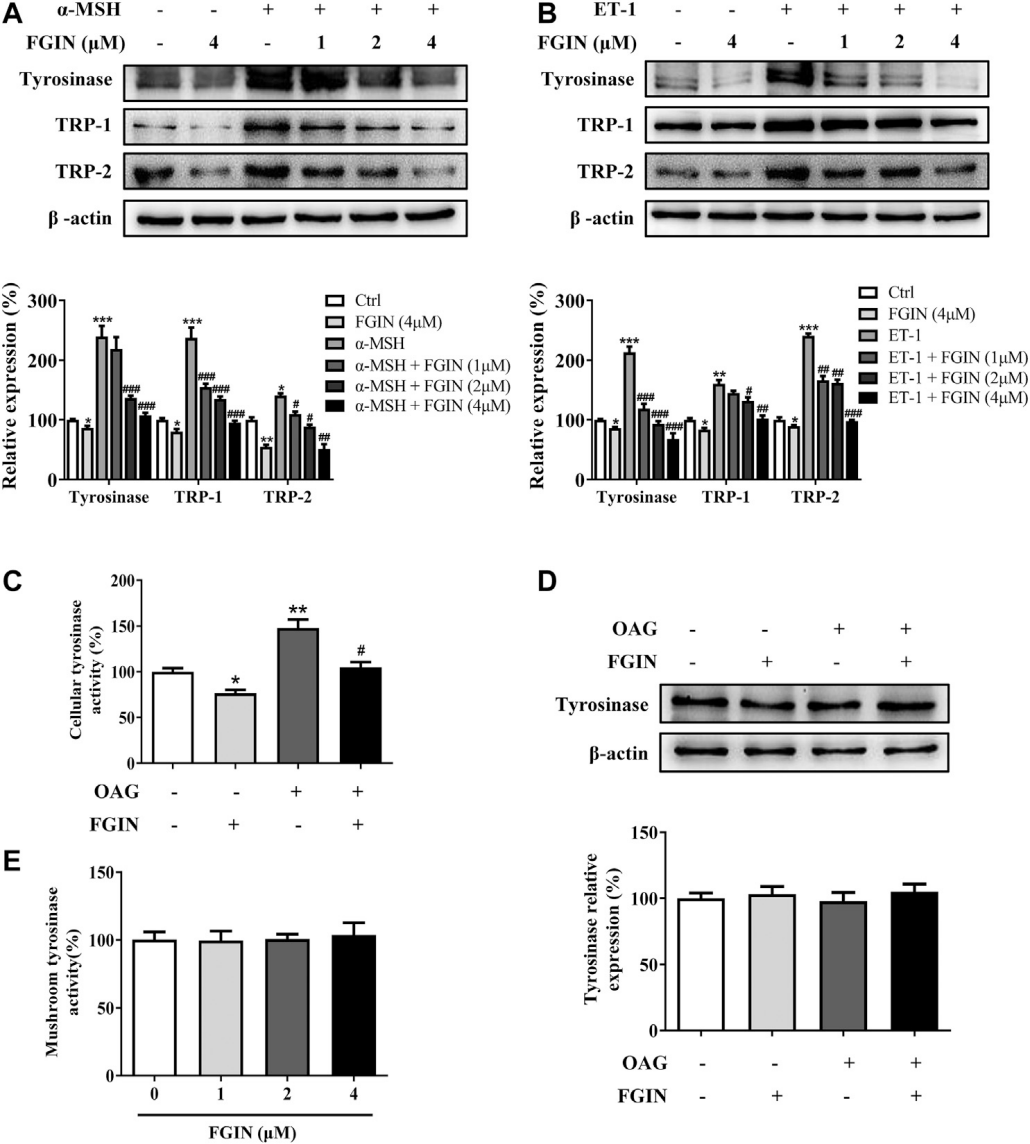
Effect of FGIN-1-27 (FGIN) on the expression of tyrosinase and the activity of tyrosinase. **(A)** SK-MEL-2 cells were treated with FGIN-1-27 (0, 1, 2, 4 μM) in the presence or absence of α-MSH (50 nM) for 48 h and western blot was then applied to detect the tyrosinase, TRP-1 and TRP-2 levels. **(B)** SK-MEL-2 cells were treated with FGIN-1-27 (0, 1, 2, 4 μM) in the presence or absence of ET-1 (10 nM) for 48 h and western blot was then applied to detect the tyrosinase, TRP-1 and TRP-2 levels. SK-MEL-2 cells were treated with FGIN-1-27 (4 μM) in the presence or absence of OAG (200 μM) for 12 h. **(C)** Cellular tyrosinase activity was determined by L-DOPA oxization as described in methods, and **(D)** western blot was applied to detect the tyrosinase levels. **(E)** Mushroom tyrosinase activity was determined as described in methods. Data are expressed as the mean ± SD (*n* = 3). *p < 0.05, **p < 0.01, ***p < 0.001 vs. non-treated cells. ^#^ *p* < 0.05, ^##^ *p* < 0.01, ^###^ *p* < 0.001 vs. α-MSH, ET-1 or OAG-treated cells.

Melanogenesis is controlled by three critical enzymes: tyrosinase, TRP-1, and TRP-2 (Zhu et al., 2015). α-MSH and ET-1 promoted melanin synthesis via increasing three crucial melanogenic enzymes expression (Regazzetti et al., 2015; Lee et al., 2013). To investigate whether the whitening effects of FGIN-1-27 is related to these proteins' expression, we studied the effect of FGIN-1-27 on the expression of tyrosinase, TRP-1, and TRP-2 via western-blot analysis. As shown in Figure 2A, FGIN-1-27 suppressed tyrosinase, TRP-1 and TRP-2 expression with or without α-MSH. FGIN-1-27 also consistently reversed ET-1-induced tyrosinase, TRP-1, and TRP-2 expression increase (Figure 2B). Unlike α-MSH and ET-1, OAG promoted melanogenesis by directly increasing cellular tyrosinase activity (D'Mello et al., 2016). As shown in Figure 2C and 2D, FGIN-1-27 treatment 12 h inhibited OAG-induced cellular tyrosinase activity increase and did not affect the expression of tyrosinase. Besides, mushroom tyrosinase activity assay was conducted to examine the direct effects of FGIN-1-27 on tyrosinase activity. As shown in Figure 2E, FGIN-1-27 did not affect the enzymatic activities of mushroom tyrosinase. These results suggested that FGIN-1-27 suppressed the expression of tyrosinase, TRP-1, TRP-2, and decreased the cellular tyrosinase activity, but had no direct effect on the enzymatic activities of tyrosinase.

### FGIN-1-27 Decreased the MITF Expression

**FIGURE 3:**
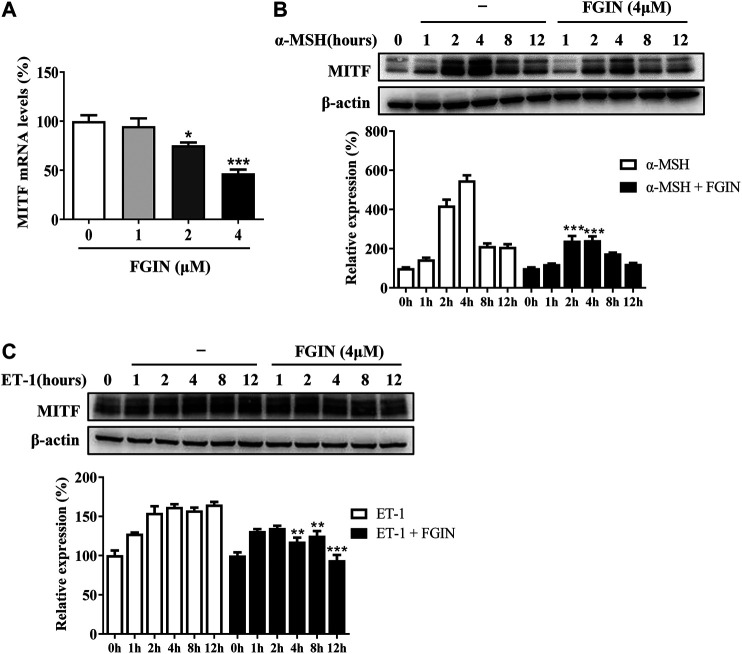
Effect of FGIN-1-27 (FGIN) on the expression of MITF in SK-MEL-2 cells. **(A)** SK-MEL-2 cells were treated with FGIN-1-27 for 4 h and RT-qPCR was then applied to detect MITF gene expression. **(B and C)** SK-MEL-2 cells were treated with α-MSH (50 nM) or ET-1 (10 nM) in the presence or absence of FGIN-1-27 for different times and western blot was then applied to detect MITF protein levels. Data are expressed as the mean ± SD (*n* = 3). *p < 0.05, **p < 0.01, ***p < 0.001 vs. α-MSH or ET-1-treated time-matched cells.

MITF is the master transcriptional factor that induces the expression of tyrosinase, TRP-1 and TRP-2 (Kawasaki et al., 2008; Levy and Fisher, 2011). As shown in Figure 3A, FGIN-1-27 inhibited the MITF transcription. We also measured the expression of MITF after SK-MEL-2 cells treated with α-MSH in the presence or absence of FGIN-1-27. As shown in Figure 3B, α-MSH significantly promoted the expression of MITF. Interestingly, the expression of MITF significantly decreased in the presence of FGIN-1-27. FGIN-1-27 also consistently suppressed the ET-1 induced MITF expression increase (Figure 3C). These results suggested that FGIN-1-27 inhibited the expression of MITF.

### FGIN-1-27 Decreased the Expression of PKC-β, p-PKA Cat, p-CREB, p-p38, and p-ERK

**FIGURE 4:**
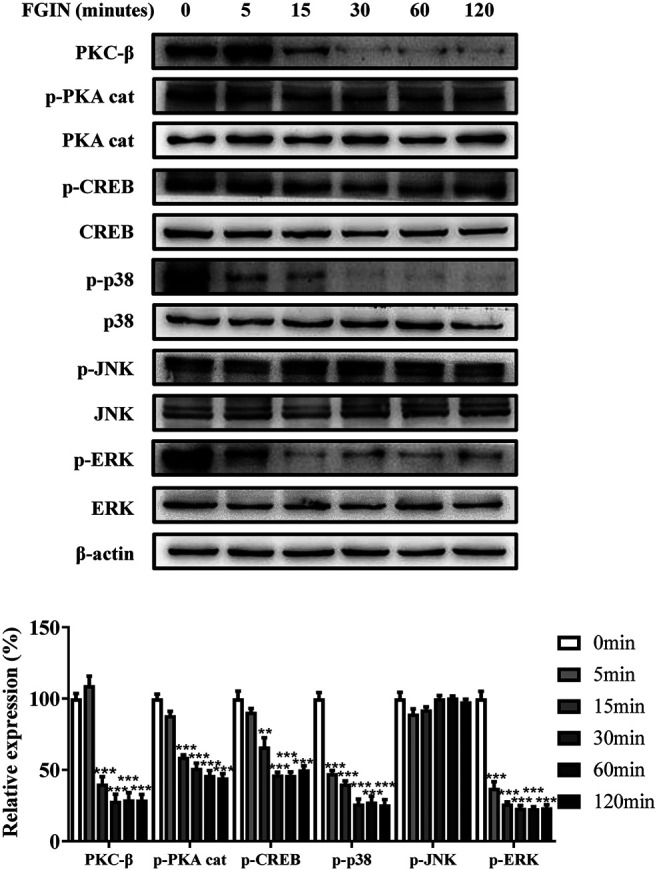
Effect of FGIN-1-27 on the activity of PKC-β, PKA/CREB and MAPK signaling pathways in SK-MEL-2 cells. SK-MEL-2 cells were treated with FGIN-1-27 (4 μM) for the indicated time period (0–120 min), and the expression of PKC-β, p-PKA cat, PKA cat, p-CREB, CREB, p-p38, p38, p-JNK, JNK, p-ERK and ERK were measured by western blot. Data are expressed as the mean ± SD (*n* = 3). **p < 0.01, ***p < 0.001 vs. non-treated cells.

PKA, PKC, and MAPK are critical transduction pathways in melanogenesis (D'Mello et al., 2016). The second messenger cAMP could activate PKA, which phosphorylates CREB, finally promoting the expression of MITF (Corre et al., 2004; Rzepka et al., 2016). The PKC-β -dependent pathway regulates melanogenesis through the activation of tyrosinase (Kim et al., 2006; Kawaguchi et al., 2012; Yuan and Jin, 2018). MAPK, including p38, extracellular signal-regulated protein kinase (ERK), and c-jun N-terminal kinase (JNK), were reported to regulate pigmentation (Zhou et al., 2014). To further understand the molecular mechanisms of melanogenesis regulation by FGIN-1-27, we measured the transduction pathways involved in the pigmentation. As shown in Figure 4, FGIN-1-27 markedly decreased the expression of PKC-β, p-PKA cat, p-CREB, p-p38, and p-ERK.

### FGIN-1-27 Inhibited Melanogenesis in Human Melanocytes

**FIGURE 5:**
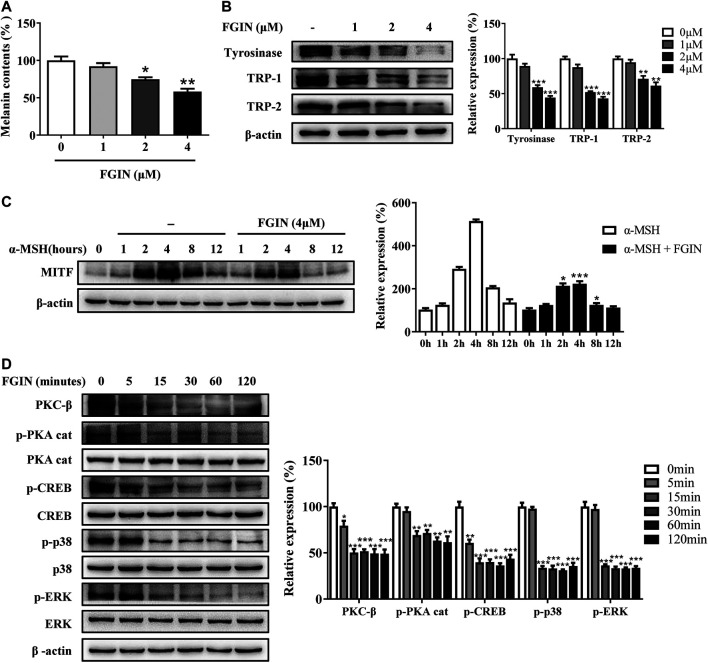
Effect of FGIN-1-27 (FGIN) on melanogenesis in human melanocytes. **(A)** Human melanocytes were treated with FGIN-1-27 for 48 h and melanin contents were measured as described in methods. **(B)** Human melanocytes were treated with FGIN-1-27 for 48 h and the expression of tyrosinase, TRP-1 and TRP-2 were measured using western-blot as described in methods. **(C)** Human melanocytes were treated with α-MSH (50 nM) in the presence or absence of FGIN-1-27 for different times and western blot was then applied to detect MITF protein levels. **(D)** Human melanocytes were treated with FGIN-1-27 for the indicated time period (0–120 min), and the expression of PKC-β, p-PKA cat, PKA cat, p-CREB, CREB, p-p38, p38, p-ERK and ERK were measured by western blot. Data are expressed as the mean ± SD (*n* = 3). *p < 0.05, **p < 0.01, ***p < 0.001 vs. non-treated cells.

The inhibition of FGIN-1-27 on melanogenesis was assessed in human *epidermis* melanocytes (HEM). As shown in Supplementary Figure S1, FGIN-1-27 had no effects on the growth of HEM at a dosing range of 1–16 μM after 48 h. As shown in Figure 5A, FGIN-1-27 significantly inhibited melanogenesis in HEM. Western blotting analysis suggested that FGIN-1-27 decreased the expression levels of tyrosinase, TRP-1, and TRP-2 in HEM (Figure 5B). As shown in Supplementary Figure S2, FGIN-1-27 treatment 12 h inhibited OAG-induced cellular tyrosinase activity increase in HEM. In addition, we measured the expression of MITF after HEM treated with α-MSH in the presence or absence of FGIN-1-27. As shown in Figure 5C, the expression of MITF significantly decreased in the presence of FGIN-1-27. Furthermore, as in SK-MEL-2 cells, the involvement of PKC-β, PKA, and MAPK in FGIN-1-27-mediated anti-melanogenic was also confirmed in HEM (Figure 5D).

### Effects of FGIN-1-27 on Pigmentation in Zebrafish

**FIGURE 6:**
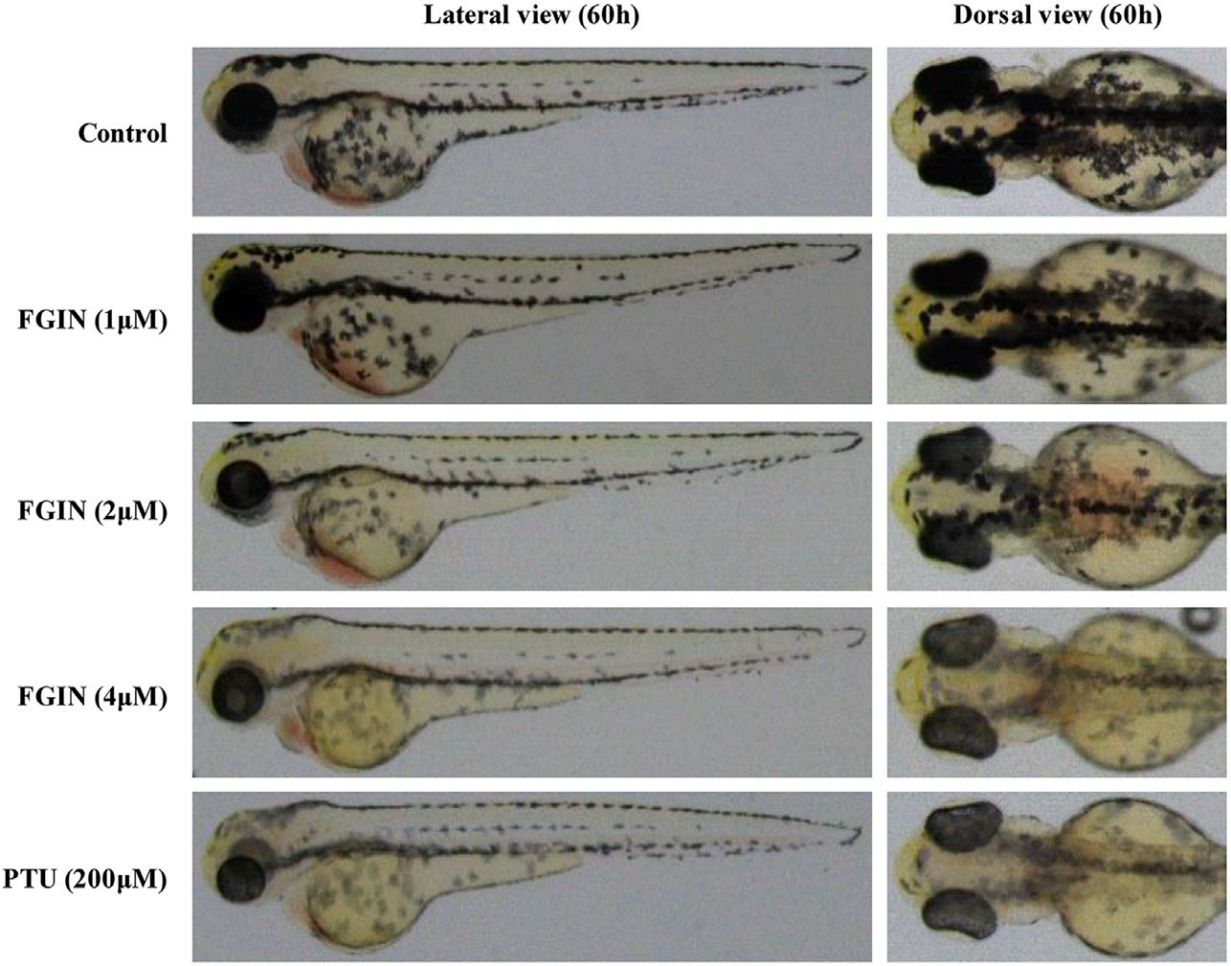
Effect of FGIN-1-27 (FGIN) on pigmentation in zebrafish. Representative photographs of zebrafish. Zebrafish embryos were treated with PTU or FGIN-1-27 from 35 to 60 h. The effects on the pigmentation of zebrafish were observed under the stereomicroscope.

Zebrafish has melanin pigments on the surface, which allows simple observation of pigmentation without complicated experimental procedures (Choi et al., 2007). PTU, a potent inhibitor of melanogenesis, which is used widely in zebrafish research (Elsalini and Rohr, 2003). In the present study, PTU was used as a positive control. As shown in Figure 6, FGIN-1-27 significantly inhibited the body pigmentation of zebrafish, similar to PTU.

### FGIN-1-27 Reduced UVB-Induced Hyperpigmentation in Guinea Pig Skin

**FIGURE 7:**
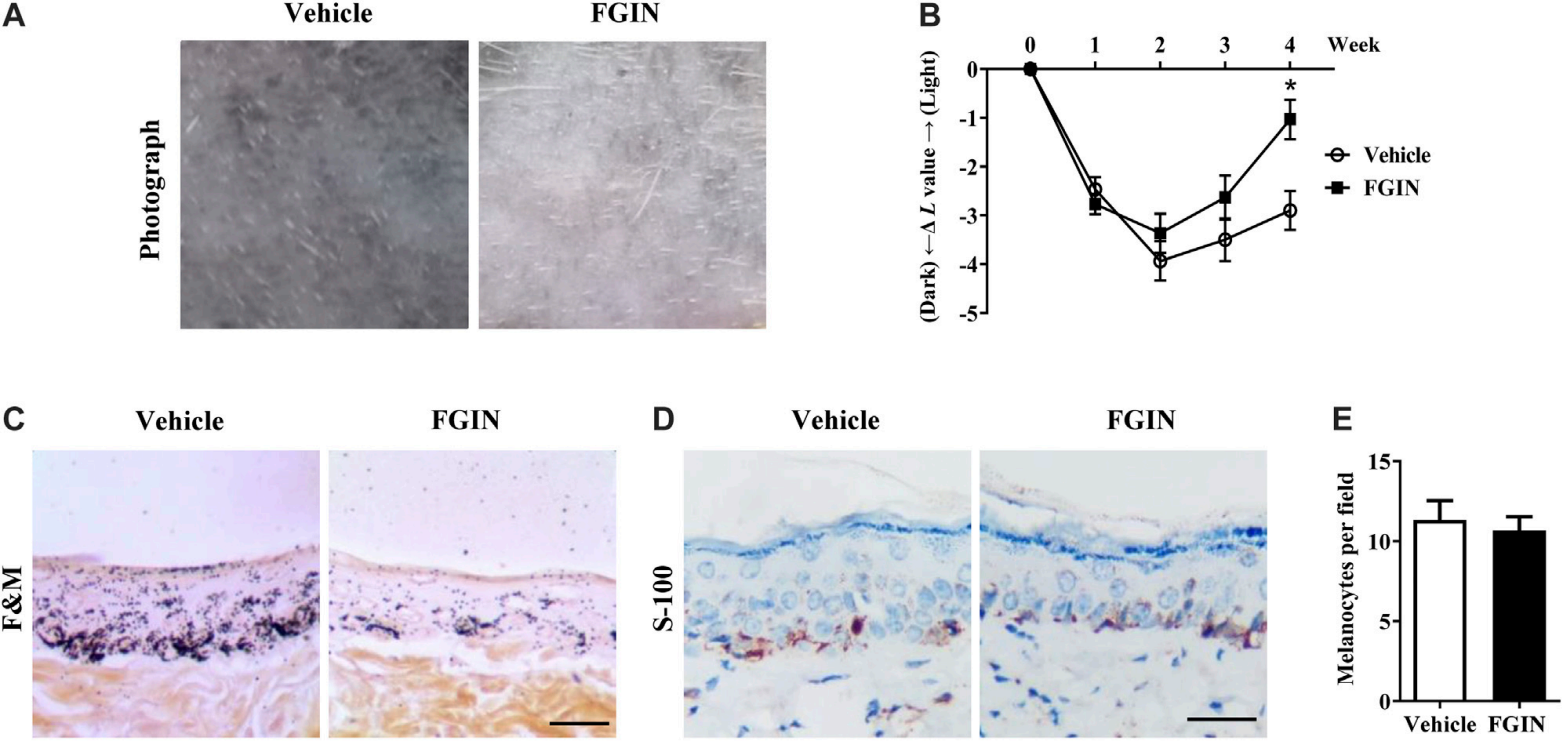
Effect of FGIN-1-27 on pigmentation in guinea-pig skin. **(A)** Representative photographs of dorsal skin of guinea pigs. **(B)** The degree of depigmentation was determined by a chromameter (CR-300; Minolta, Osaka, Japan) once a week for 4 weeks. The Δ*L* value was calculated using the *L* value (brightness index) measured with the chromameter follows: Δ*L* = *L* (at each week measured) − *L* (at day 0). Negative Δ*L* values indicate an UV-induced darkening of the skin. An increase in the Δ*L* value indicates a decrease in hyperpigmentation induced by UV. **(C)** Masson–Fontana ammoniacal silver staining of skin biopsies. **(D)** Immunohistochemical staining of skin biopsies for the detection of S-100 as a melanocyte marker protein **(E)** Number of melanocytes per microscopic field in skin sections. Bar = 50 μm *p < 0.05 vs. vehicle-treated groups.

UVB-induced hyperpigmentation model in brown guinea pigs was used to examine the whitening effect of FGIN-1-27 *in vivo*. As shown in Figure 7A, representative photographs of guinea pig skin indicated that FGIN-1-27 (1%) significantly suppressed pigmentation when compared with the vehicle treatment. To further assess the degree of pigmentation, we checked the *L* value (brightness index) using a Spectrophotometer. The Δ*L* value of FGIN-1-27 group was markedly higher than that of the vehicle group after 3 weeks treatment, suggesting that FGIN-1-27 reduced UVB-induced hyperpigmentation in guinea pig skin (Figure 7B). Masson–Fontana ammoniacal silver staining of skin tissue showed that FGIN-1-27 significantly inhibited UVB-induced pigmentation in the epidermal basal layer (Figure 7C). Immunohistochemical staining of a melanocyte marker protein, S-100, revealed that melanocyte count was not affected by FGIN-1-27 (Figures 7D,E). These results demonstrate that FGIN-1-27 has whitening effects on UV-induced hyperpigmentation *in vivo*.

## Discussion

According to the Global Industry Analysts, the global whitening market will reach $31.2 billion by 2024 (Kim et al., 2019). Many research groups are focusing their efforts to elucidate novel and effective whitening compounds. Although plenty of agents have been developed, only a few were demonstrated to be therapeutically effective due to cytotoxicity and weak efficacy (Kim et al., 2008; Singh et al., 2016). Thus, it is necessary to continue to discover more efficient and safer skin-whitening agents.

In the current study, FGIN-1-27 inhibited the basal melanogenesis and reversed α-MSH, OAG, or ET-1-induced melanin increase, without affecting cell viability (Figures 1 and 5). Tyrosinase, TRP-1, and TRP-2 are the key enzymes in melanogenesis, while α-MSH and ET-1 promote pigmentation by increasing the expression of these three crucial melanogenic enzymes (Rzepka et al., 2016; Corre et al., 2004; Regazzetti et al., 2015). Our results suggested that FGIN-1-27 suppressed α-MSH or ET-1-induced tyrosinase, TRP-1, and TRP-2 expression increase (Figures 2A,B). Tyrosinase activity is critical to melanogenesis (Rzepka et al., 2016). 1-Oleoyl-2-acetyl-sn-glycerol (OAG) is a synthetic, membrane-permeable diacylglycerol (DAG) analog demonstrated to increase the activity of tyrosinase (Thébault et al., 2005). Interestingly, FGIN-1-27 markedly inhibited OAG-induced tyrosinase activity increase, and the expression of tyrosinase did not significantly change after 12 h of treatment (Figures 2C,D). Mushroom tyrosinase activity assay showed that FGIN-1-27 did not directly inhibit tyrosinase activity, which suggested that FGIN-1-27 was not a direct inhibitor of tyrosinase (Figure 2E). Microphthalmia-associated transcription factor (MITF) is a master transcription factor for melanogenesis and upregulates the expression of tyrosinase, TRP-1, and TRP-2 (Levy and Fisher, 2011; Kawasaki et al., 2008). The present studies showed that FGIN-1-27 suppressed basal, α-MSH, and ET-1-induced MITF expression increase (Figure 3). As mentioned above, FGIN-1-27 inhibiting melanogenesis by decreasing the expression of MITF, tyrosinase, TRP-1, TRP-2 and inhibiting the tyrosinase activity, which is contradictory with previous studies that suggested MDR activation can increase melanogenesis (Lv et al., 2019). There are two possible explanations for this effect. Given the opposing functional activities of FGIN-1-27 vs. diazepam, we speculate that FGIN-1-27 is an MDR inverse agonist rather than generally considering as an agonist in melanocytes. Furthermore, many mechanisms are also involved in the effect of FGIN-1-27-at times, perhaps, obscuring the role of MDR activation. Further comprehensive studies are needed to disclose the function and underlying mechanism of the FGIN-1-27 and MDR in melanocytes.

Excessive UV irradiation is considered to be an important cause of skin darkening (Abdel-Naser et al., 2003). After exposure to UV radiation, keratinocytes and melanocytes were activated and produced α-melanocyte-stimulating hormone (α-MSH), diacylglycerol (DAG), and endothelin-1 (ET-1) (D'Mello et al., 2016; Bae-Harboe and Park, 2012). α-MSH, ET-1, and DAG affect melanin synthesis through intracellular signaling pathways. When α-melanocyte-stimulating hormone (α-MSH) binds to melanocortin-1 receptor (MC1R), the intracellular level of cAMP is elevated and the PKA/CREB pathway is activated, finally promoting melanogenesis (Corre et al., 2004; Rzepka et al., 2016). OAG could activate the PKC-β, which phosphorylates serine residues on the cytoplasmic domain of tyrosinase and activates it (Kim et al., 2006; Kawaguchi et al., 2012; Yuan and Jin, 2018). MAPK signaling pathway including extracellular p38, ERK, and JNK, could regulate melanin synthesis (Zhou et al., 2014). The activation of the p38 signaling pathway decreased the expression of MITF and promotes melanogenesis (Hirata et al., 2007). The role of ERK and JNK pathway in melanogenesis remains controversial (Lee et al., 2013; Peng et al., 2014). Endothelin-1 (ET-1) was reported to induce melanogenesis via the activation of ERK and p38 (Park et al., 2015; Regazzetti et al., 2015). Besides, the cross-talk between PKA and PKC-β could amplify the melanogenic effect and MAPK provides meeting points for cross-talk between these signaling pathways (Lee and Noh, 2013). A great deal of attention has continuously focused on the development of novel skin-whitening agents, which inhibited melanogenesis by regulating above pigmentation-related signaling pathways. Kim et al. research indicated that piperlonguminine inhibited PKA/CREB-mediated melanogenesis but did not affect PKC-mediated melanogenesis (Kim et al., 2006). Furthermore, haginin A decreased melanogenesis via affecting ERK pathways, but had no effects on PKA/CREB pathways (Fujimoto et al., 1988). In the present study, FGIN-1-27 decreased the expression of PKC-β, p-PKA cat, p-CREB, p-p38 and p-ERK (Figures 4 and 5). These results suggested that all three of the above-mentioned signaling pathways involve melanogenesis was inhibited after FGIN-1-27 treated. This can explain why FGIN-1-27 inhibited α-MSH, ET-1, or OAG-induced melanogenesis.

Furthermore, we investigated the effects of FGIN-1-27 on melanogenesis of zebrafish. Zebrafish is a highly beneficial vertebrate model organism because its organ system and gene sequence are similar to those of humans (Choi et al., 2007). Furthermore, zebrafish has melanin pigments on the surface, which allows simple observation of pigmentation without complicated experimental procedures (Kim et al., 2008). In the present study, FGIN-1-27 significantly decreased the body pigmentation in zebrafish (Figure 6), which is contradictory with previous studies that suggested MDR activation slightly increased the number of melanocytes in larval zebrafish (Allen et al., 2020). The possible reason is that there exists a different mechanism in the anti-melanogenic effect of FGIN-1-27. Further comprehensive research is needed to elicit the role of FGIN-1-27 in melanogenesis and melanocyte production in zebrafish. We also investigated the effects of FGIN-1-27 on melanogenesis in the skin of guinea pigs. As shown in Figure 7, we found that the topical application of FGIN-1-27 to the dorsal skin of guinea pig in which hyperpigmentation had been induced by exposure to UVB, resulted in efficient whitening effects. These results suggested that FGIN-1-27 inhibited melanin production in active melanocytes, but not a reduction of numbers of melanocytes.

**FIGURE 8:**
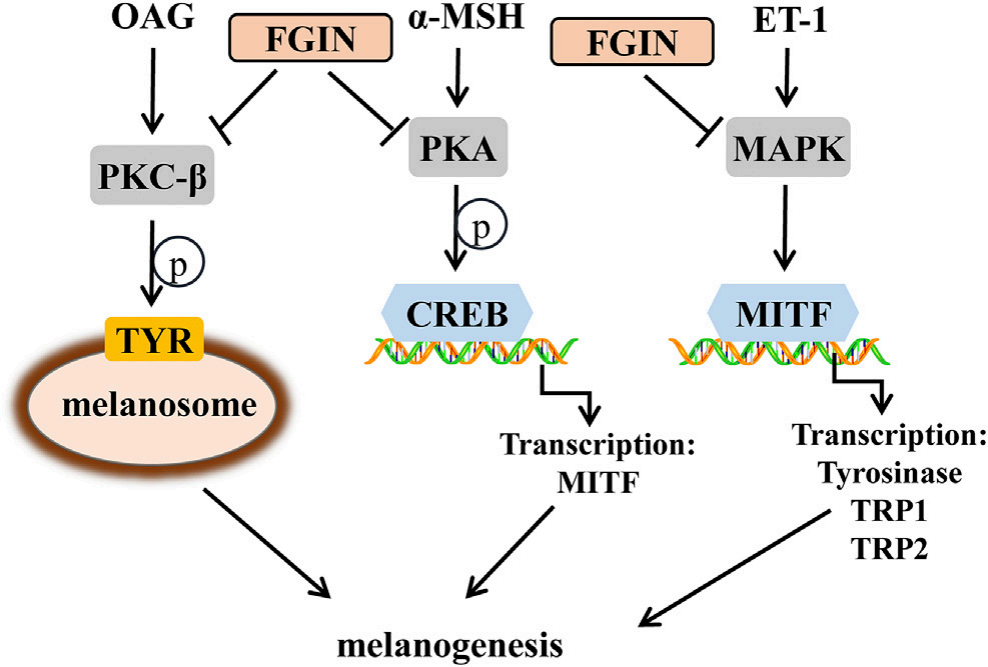
Schematic description of changes in pigmentation upon FGIN-1-27(FGIN) treatment.

In conclusion, our results demonstrated that FGIN-1-27 exerted anti-melanogenic effects, as well as the mechanisms responsible for these effects. FGIN-1-27 induced anti-melanogenic effects in melanocytes by suppressing PKA/CREB, PKC-β and MAPK pathways, which ultimately results in the inhibition of tyrosinase expression and activity (Figure 8). During *in vivo* experiments, FGIN-1-27 inhibited the body pigmentation of zebrafish and reduced UVB-induced hyperpigmentation in guinea pig skin. Compounded with the fact that FGIN-1-27 exhibited no cytotoxic activity in our research, it suggested that FGIN-1-27 may be effective as a safer skin-whitening agent.

## Data Availability Statement

The original contributions presented in the study are included in the article/Supplementary Material, further inquiries can be directed to the corresponding author.

## Author Contributions

JL, YC, and GS conceived and designed the study, provided critical comments and edited the manuscripts. SJ and YY carried out major experiments. XZ and RG performed analysis and interpretation of data on immunoblot analysis assay. JL performed on data collecting. All authors read and approved the final manuscript.

## Funding

This study was sponsored by the Fund of Changzhou Sci&Tech Program (Grant No. CJ20180007) to JL.

## Conflict of Interest

The authors declare that the research was conducted in the absence of any commercial or financial relationships that could be construed as a potential conflict of interest.
