# Supplementary material for: FGIN-1-27 Inhibits Melanogenesis by Regulating Protein Kinase A/cAMP-Responsive Element-Binding, Protein Kinase C-β, and Mitogen-Activated Protein Kinase Pathways
Source: Front Pharmacol. 2020 Dec 3;11:602889. doi: 10.3389/fphar.2020.602889 (PMC7775666; doi:10.3389/fphar.2020.602889)
Supplement: Supplementary file 1 [file datasheet1.pdf]

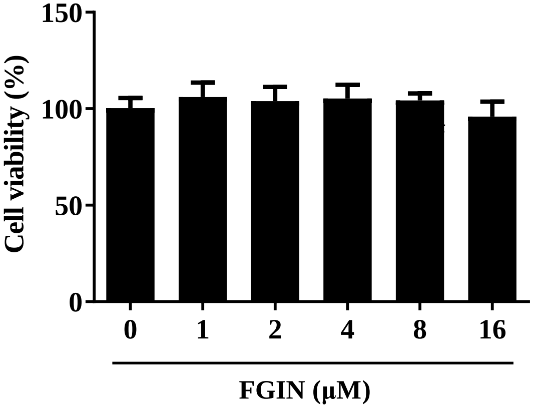

Figure S1. Effect of FGIN-1-27 (FGIN) on cell viability in human melanocytes. After incubation of with various concentrations (1-16 μM) of FGIN-1-27 for 48 h, cell viability was determined using MTT assay. Data are expressed as the mean ± SD (n=3).

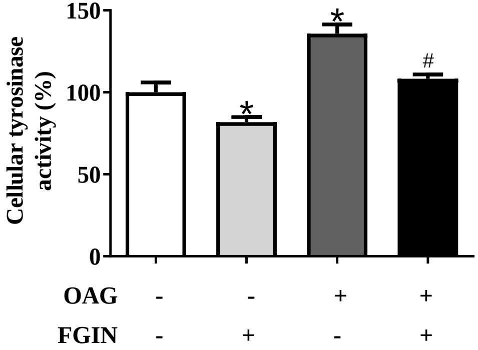

Figure S2. Effect of FGIN-1-27 (FGIN) on the tyrosinase activity in human melanocytes. Human melanocytes were treated with FGIN-1-27 (4μM) in the presence or absence of OAG (200 μM) for 12 h. Cellular tyrosinase activity was determined by L-DOPA oxization as described in methods. Data are expressed as the mean ± SD (n=3). *p<0.05 versus non-treated cells. #p<0.05 versus OAG-treated cells.
