# Supplementary material for: FGIN-1-27 Inhibits Melanogenesis by Regulating Protein Kinase A/cAMP-Responsive Element-Binding, Protein Kinase C-β, and Mitogen-Activated Protein Kinase Pathways
Source: Front Pharmacol. 2020 Dec 3;11:602889. doi: 10.3389/fphar.2020.602889 (PMC7775666; doi:10.3389/fphar.2020.602889)
Supplement: Supplementary file 2 [file datasheet2.pdf]

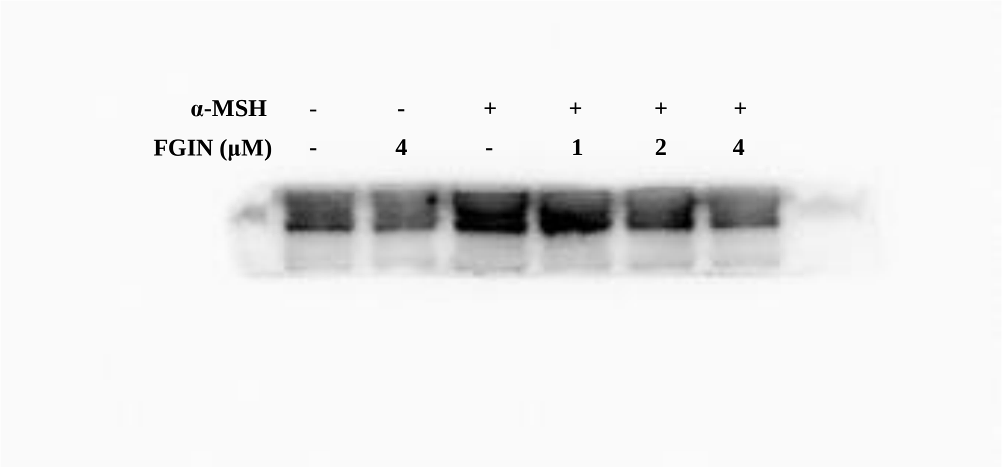

Figure 2A Tyrosinase

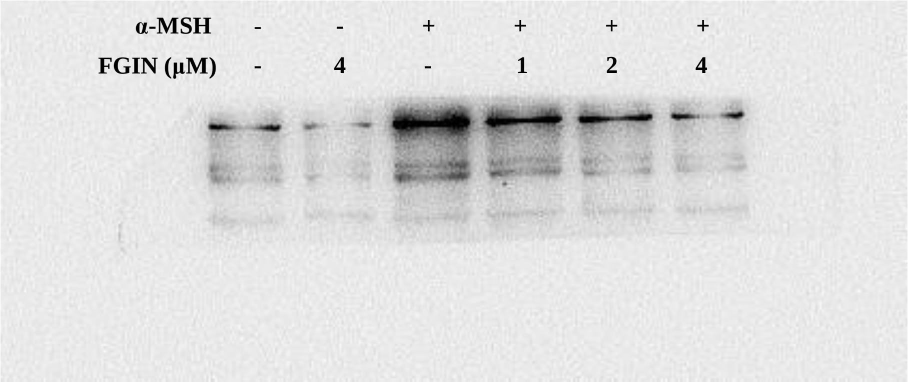

Figure 2A TRP-1

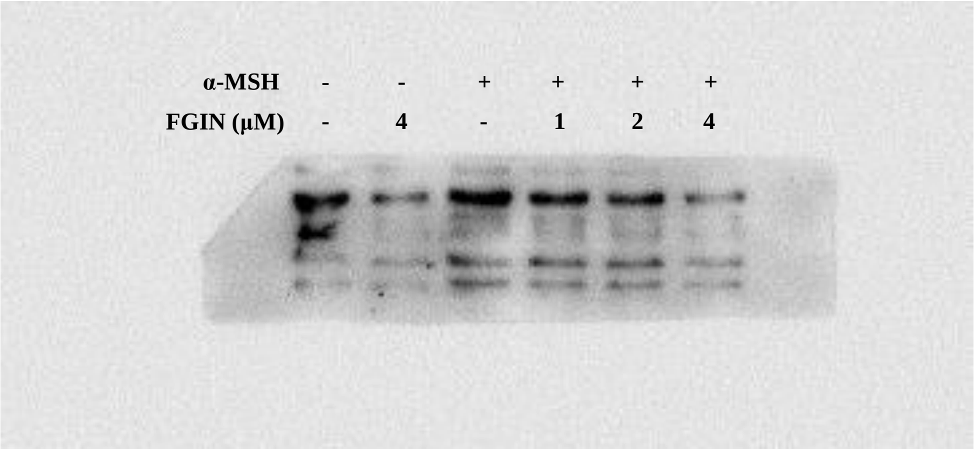

Figure 2A TRP-2

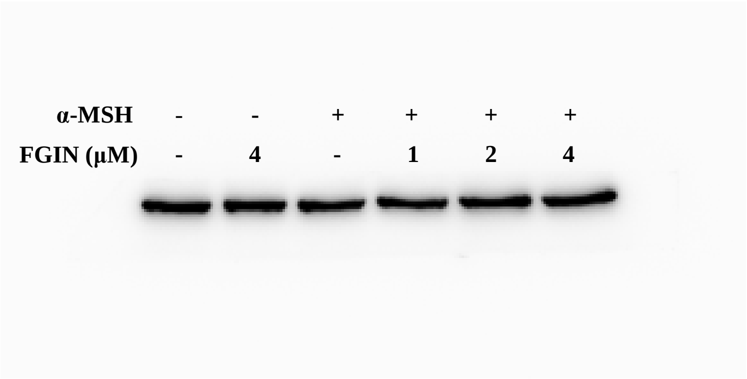

Figure 2A β-actin

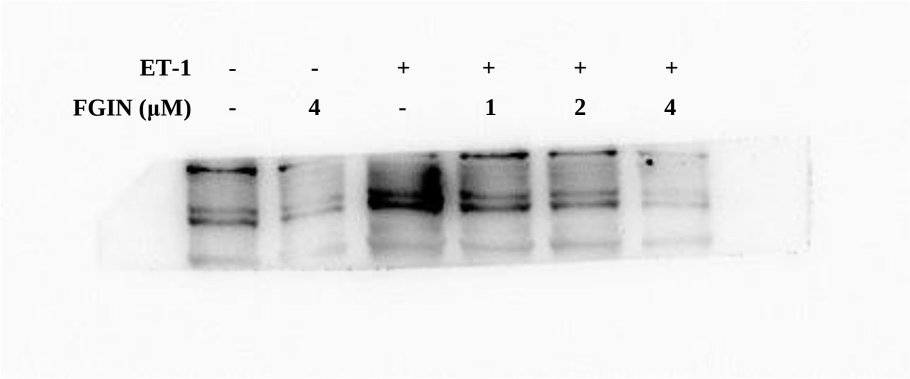

Figure 2B Tyrosinase

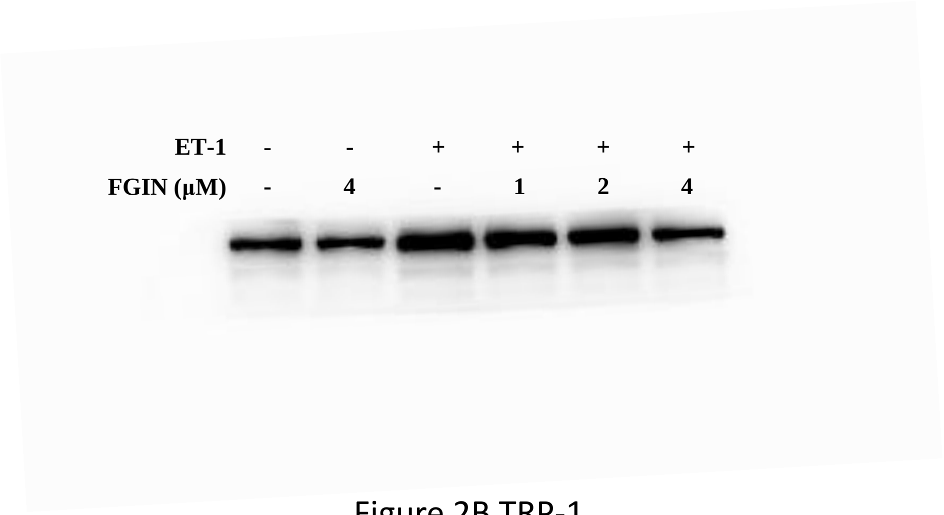

Figure 2B TRP-1

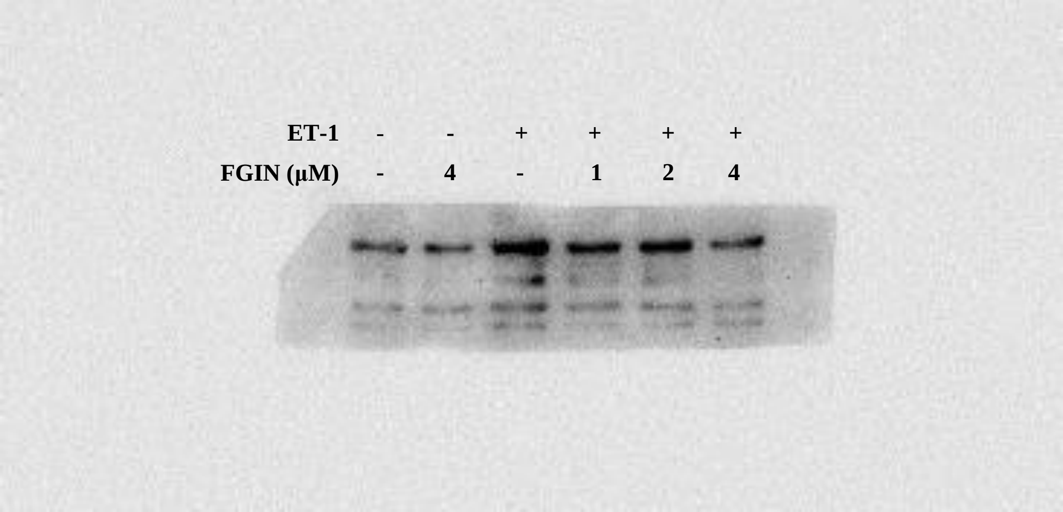

Figure 2B TRP-2

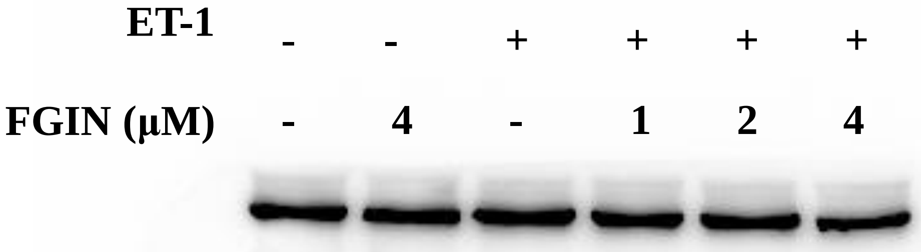

Figure 2B β-actin

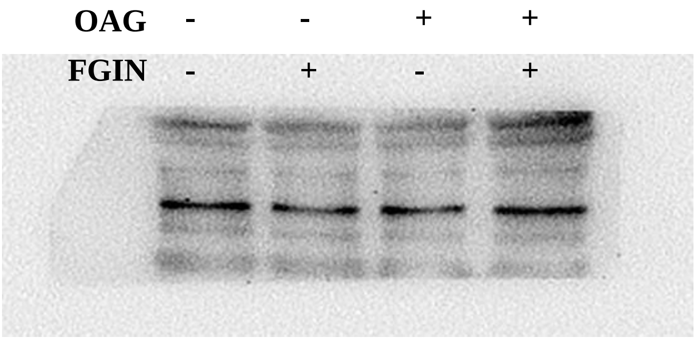

Fig. 2d Tyrosinase

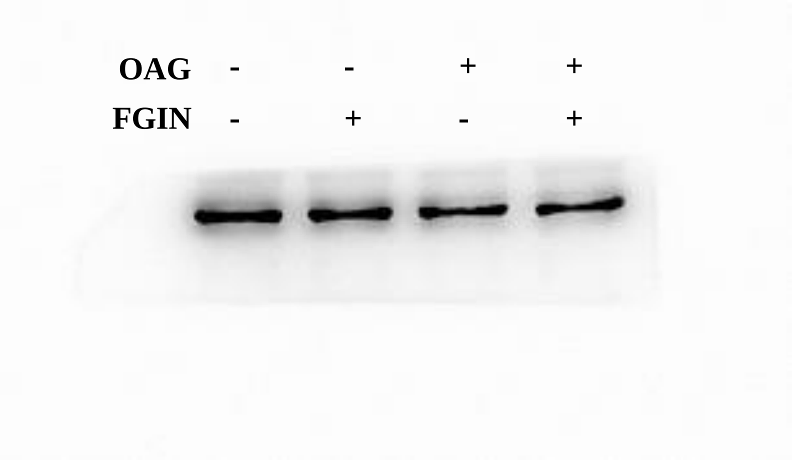

Fig. 2d β-actin

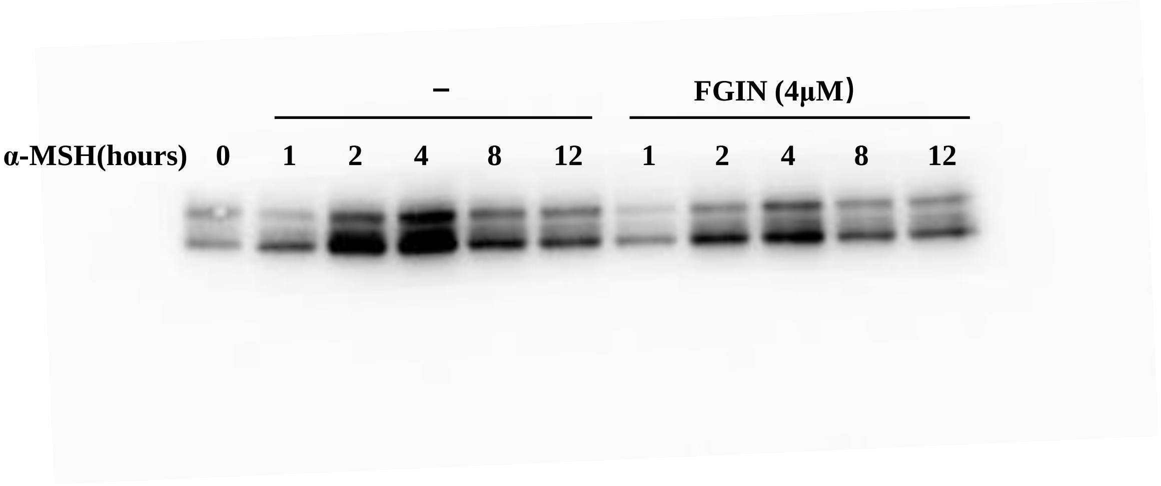

Figure 3B MITF

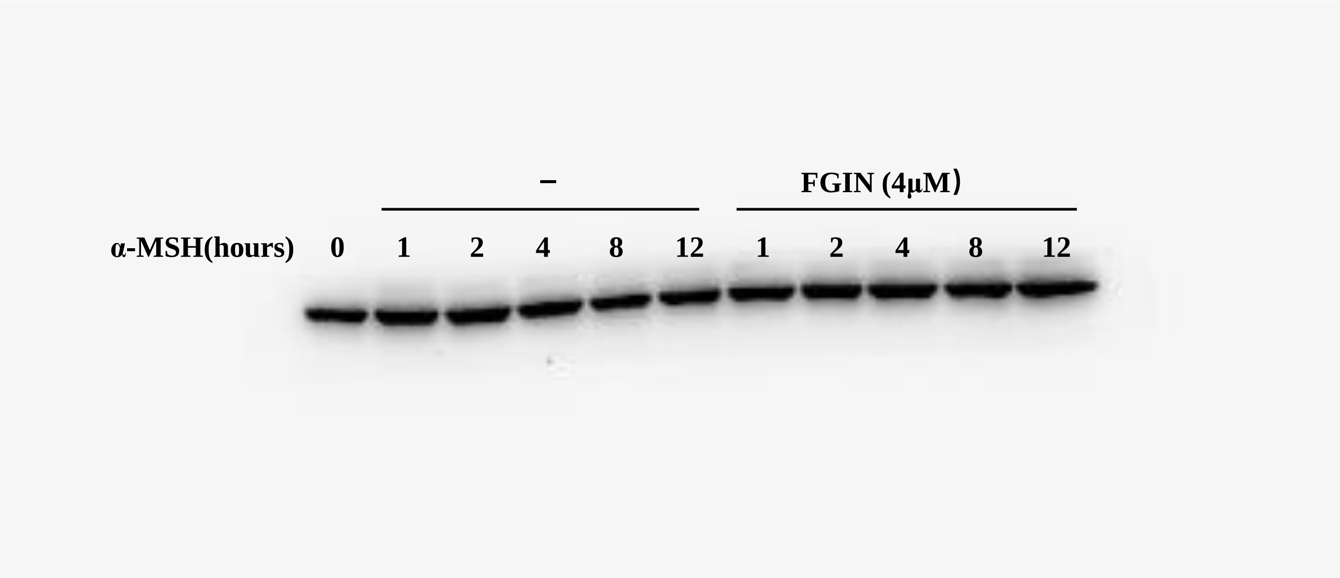

Figure 3B β-actin

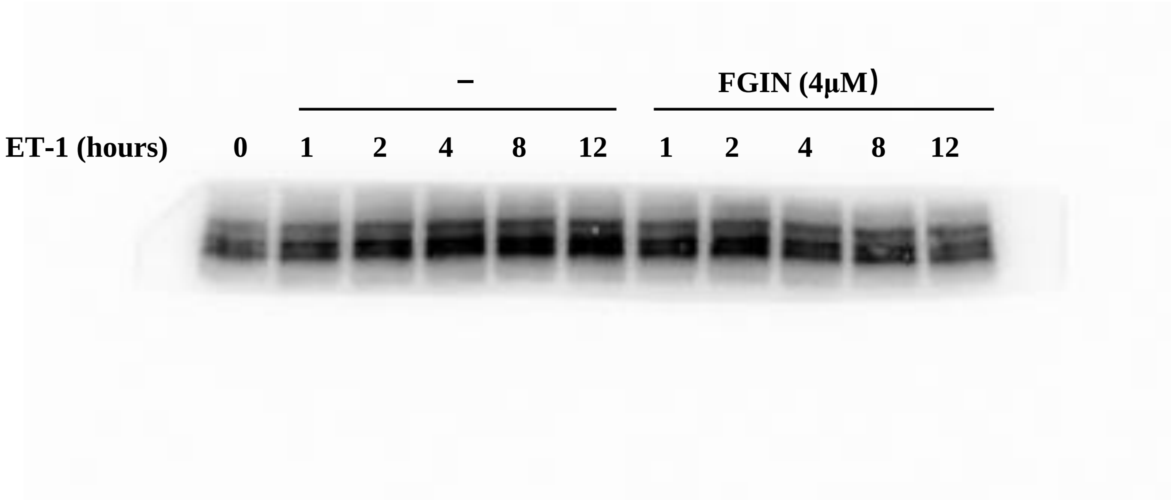

Figure 3C MITF

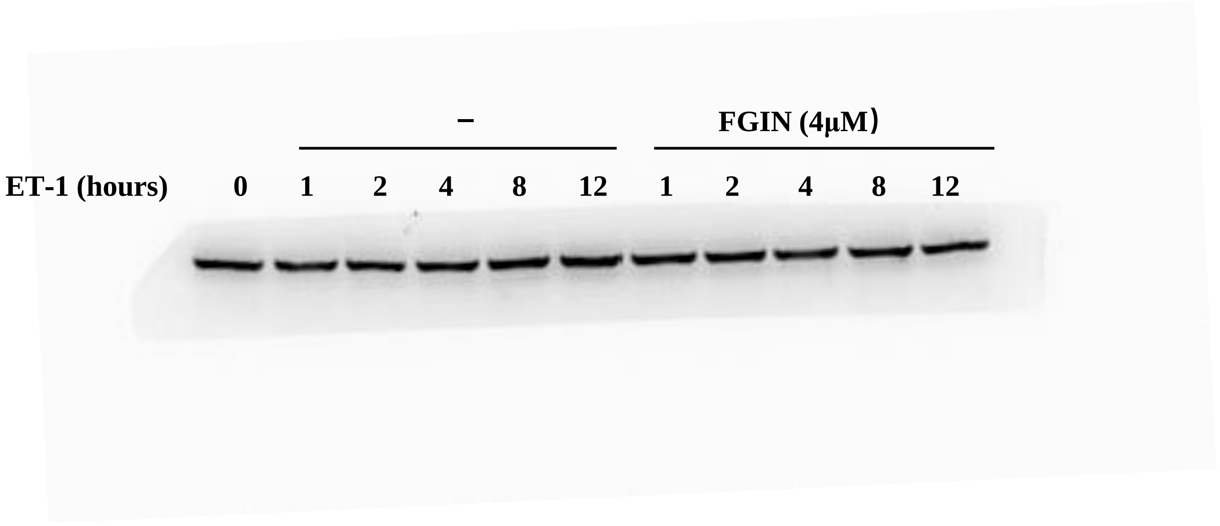

Figure 3C β-actin

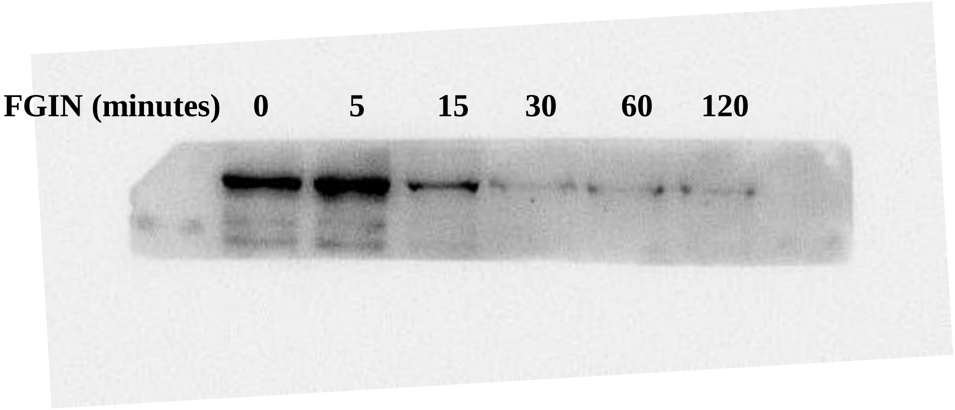

Figure 4 PKC-β

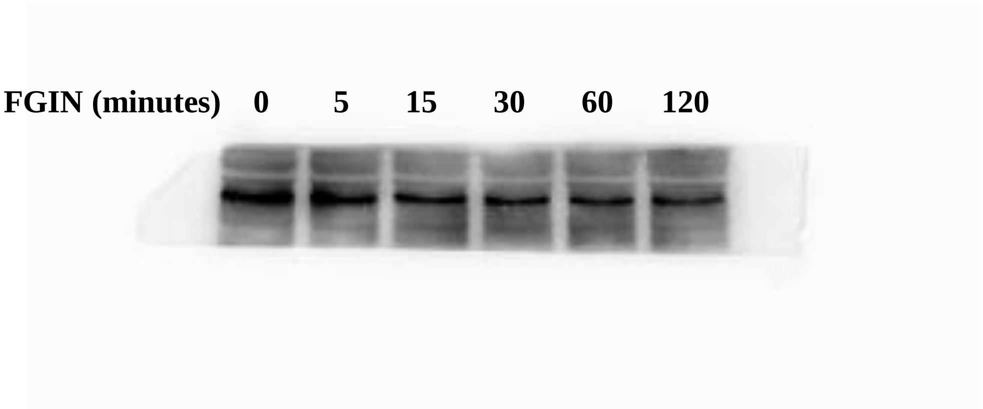

Figure 4 p-PKA cat

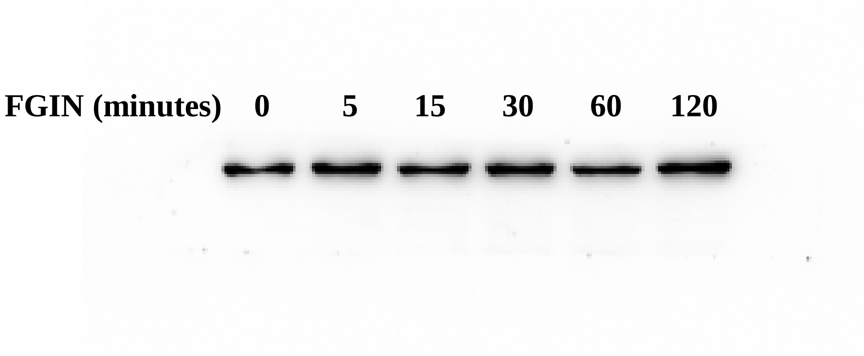

Figure 4 PKA cat

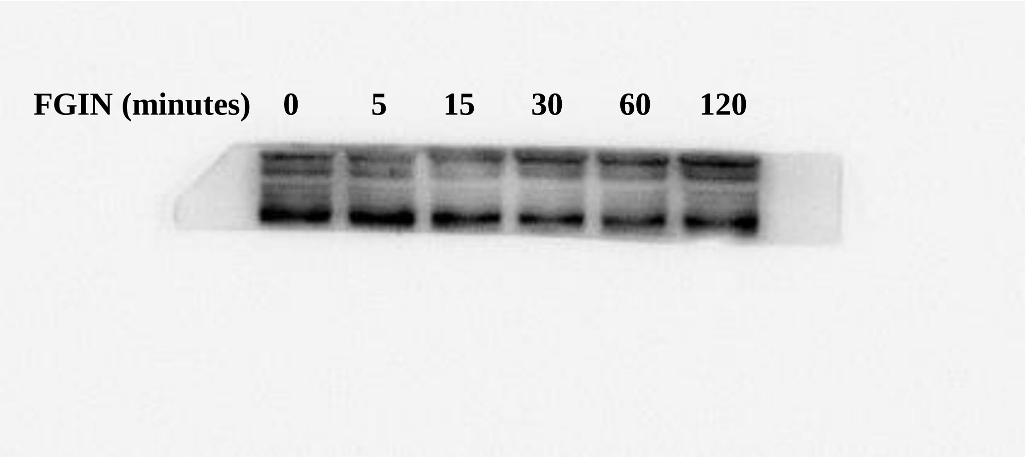

Figure 4 p-CREB

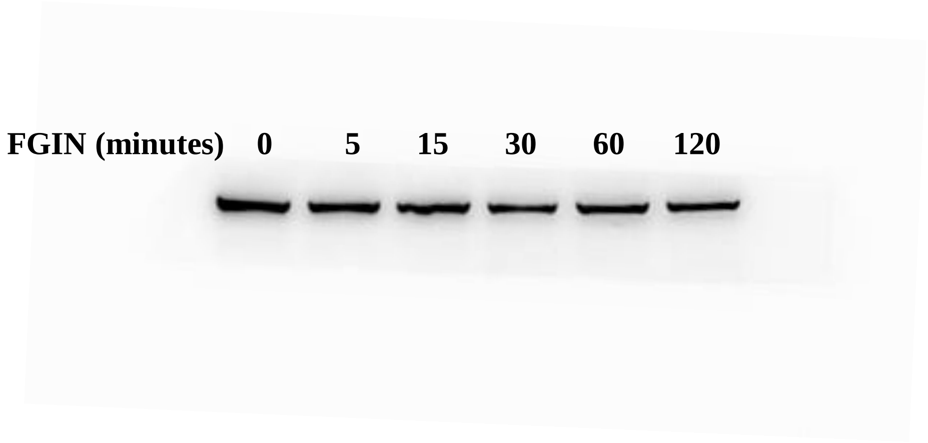

Figure 4 CREB

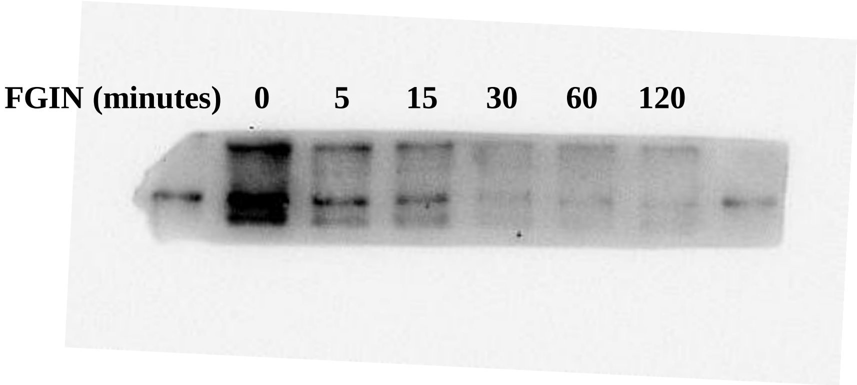

Figure 4 p-p38

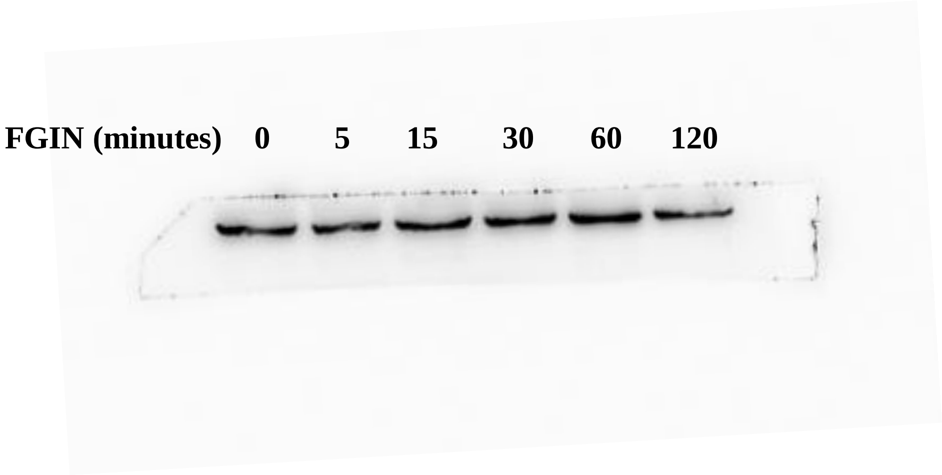

Figure 4 p38

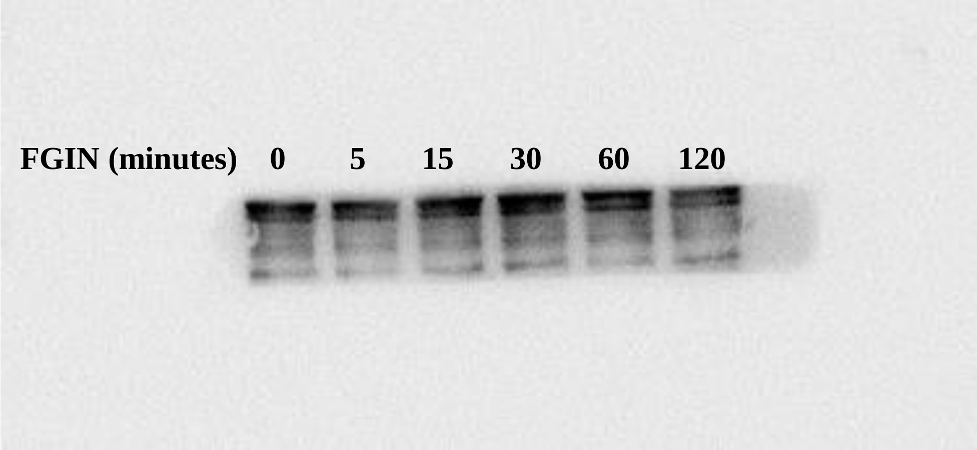

Figure 4 p-JNK

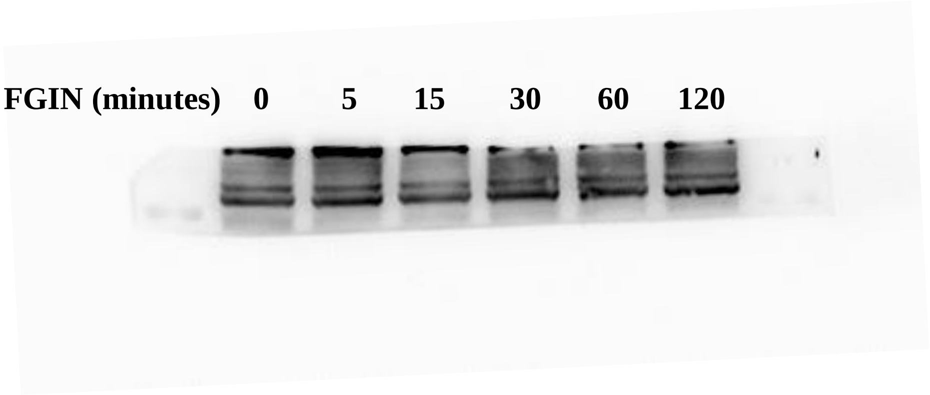

Figure 4 JNK

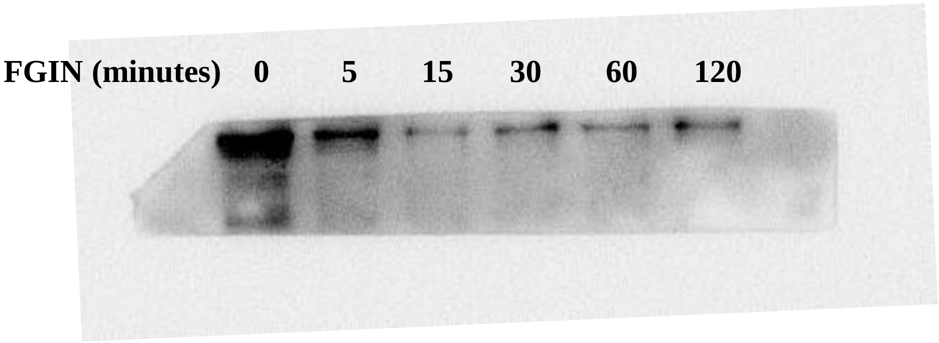

Figure 4 p-ERK

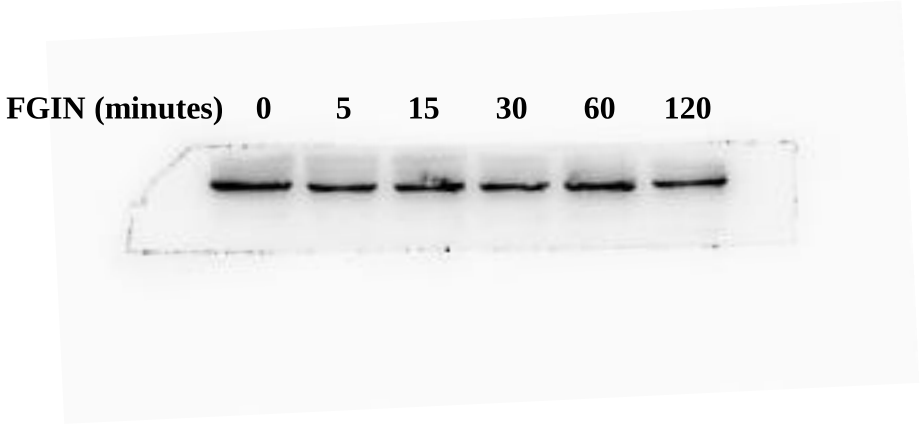

Figure 4 ERK

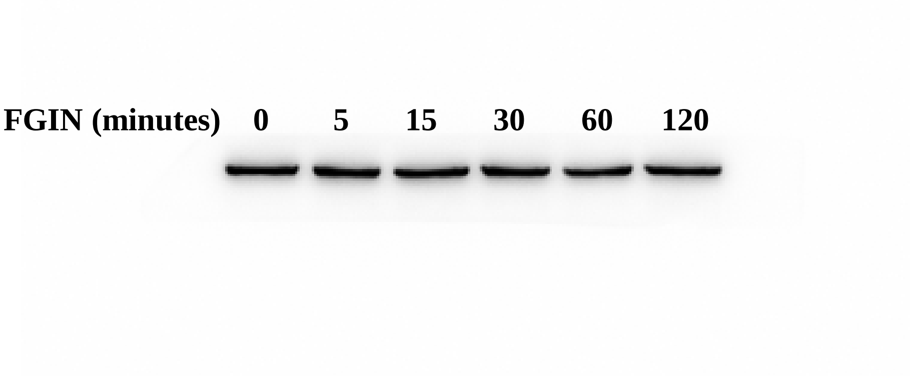

Figure 4 β-actin

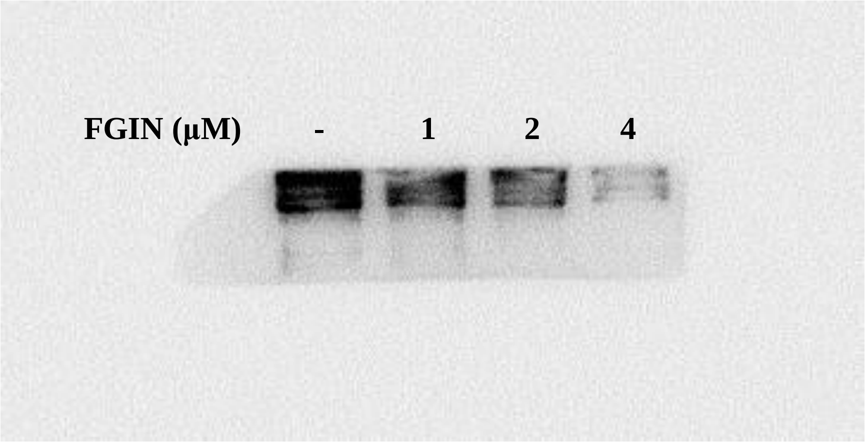

Figure 5B Tyrosinase

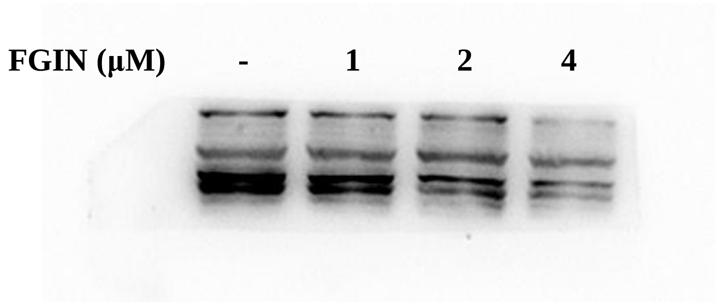

Figure 5B TRP-1

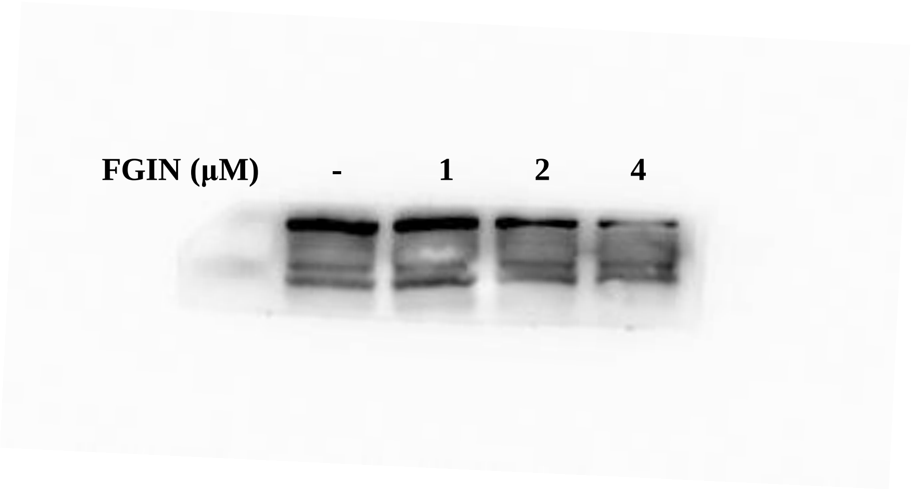

Figure 5B TRP-2

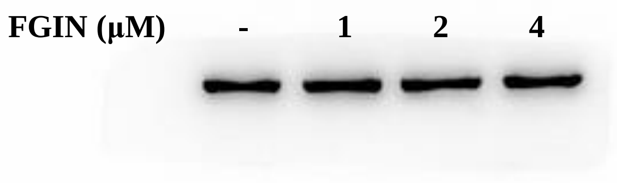

Figure 5B β-actin

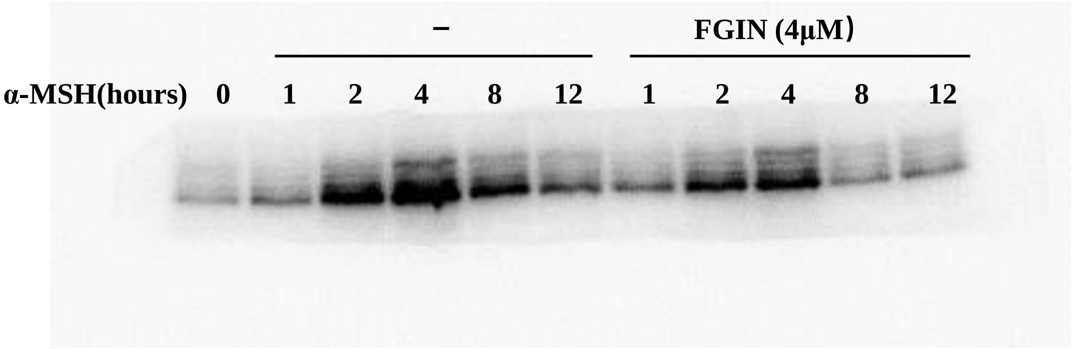

Figure 5C MITF

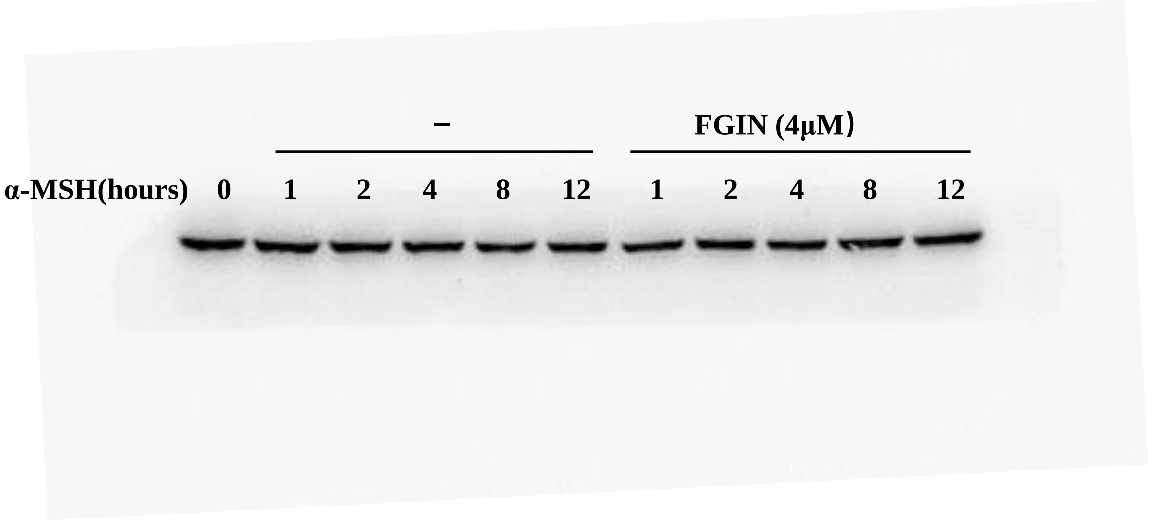

Fig. 5c β-actin

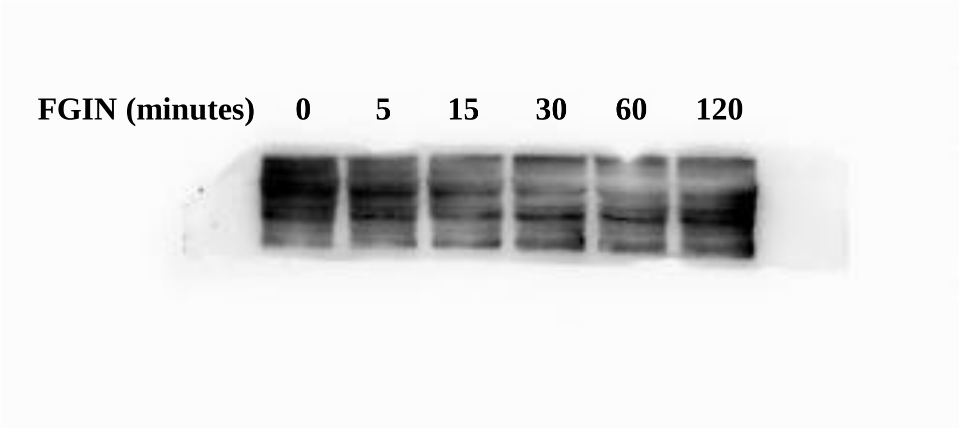

Figure 5D PKC-β

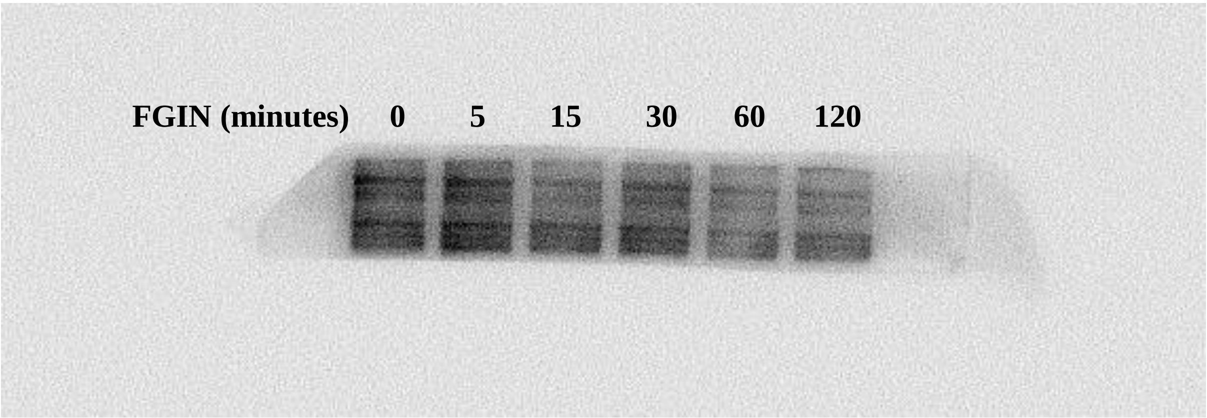

Figure 5d p-PKA cat

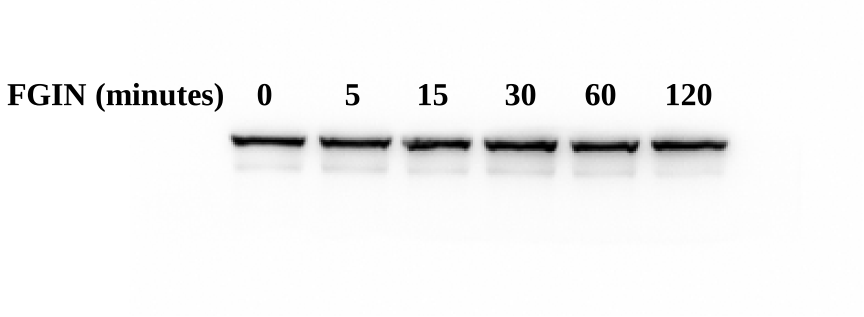

Figure 5D PKA cat

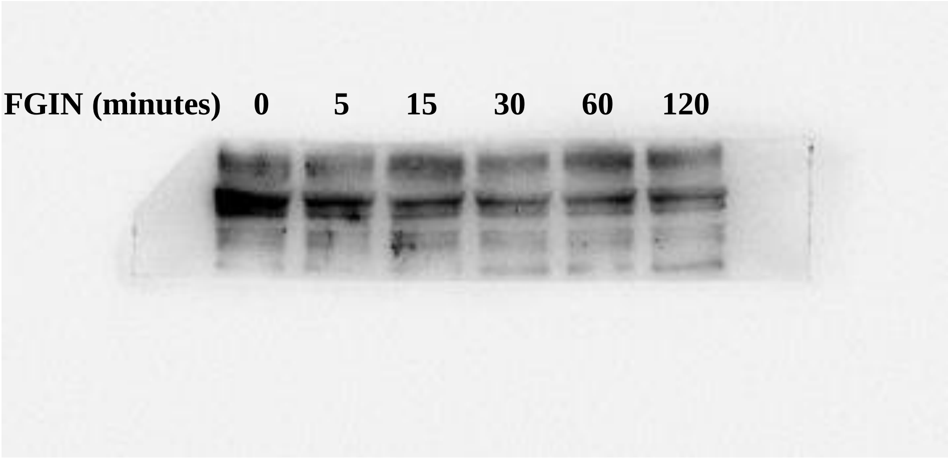

Figure 5D p-CREB

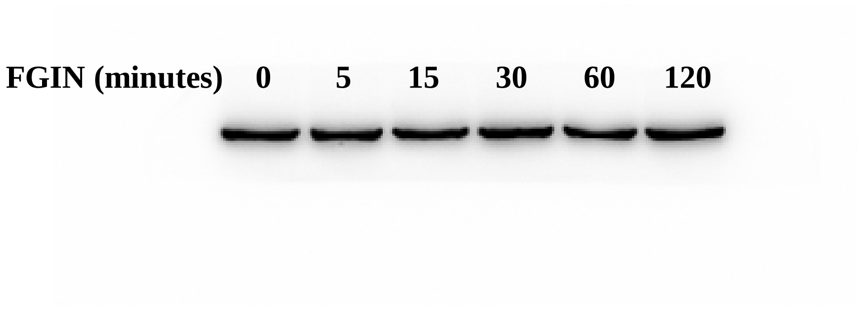

Figure 5D CREB

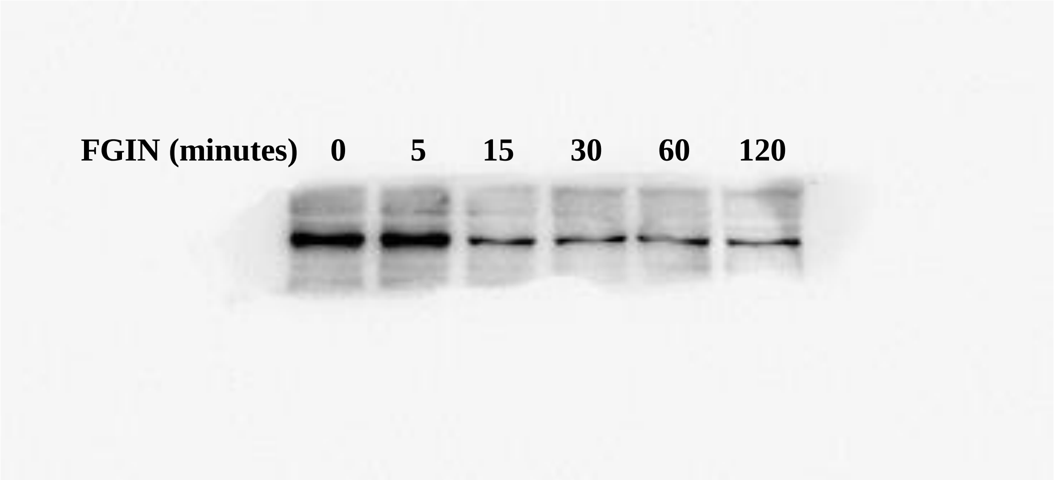

Figure 5D p-p38

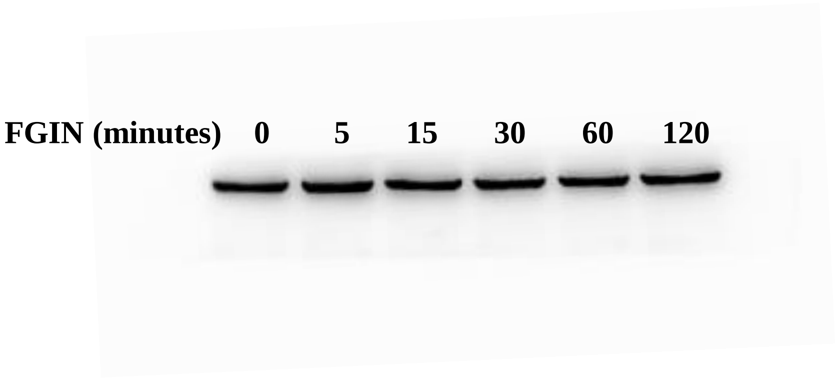

Figure 5D p38

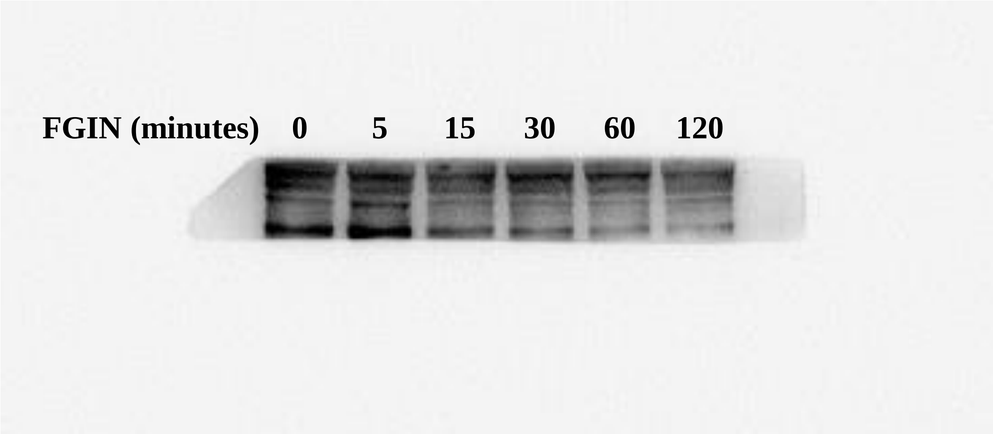

Figure 5D p-ERK

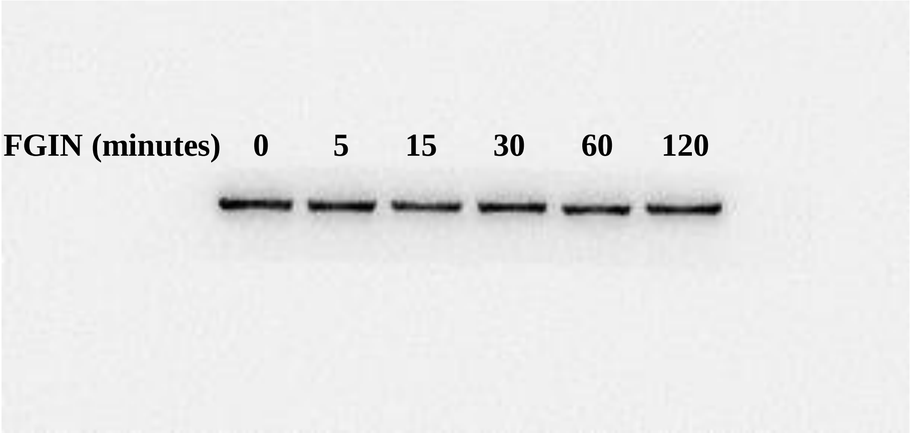

Figure 5D ERK

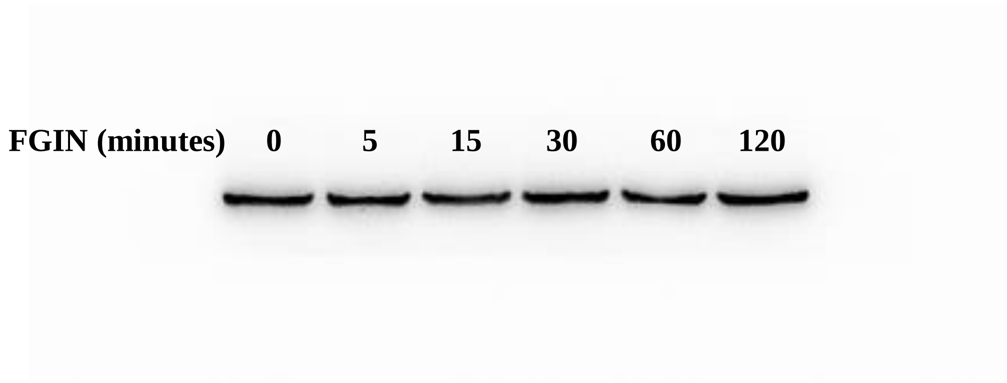

Figure 5D β -actin
